# A Novel Protein LZTFL1 Regulates Ciliary Trafficking of the BBSome and Smoothened

**DOI:** 10.1371/journal.pgen.1002358

**Published:** 2011-11-03

**Authors:** Seongjin Seo, Qihong Zhang, Kevin Bugge, David K. Breslow, Charles C. Searby, Maxence V. Nachury, Val C. Sheffield

**Affiliations:** 1 Department of Pediatrics, University of Iowa College of Medicine, Iowa City, Iowa, United States of America; 2 Howard Hughes Medical Institute, Chevy Chase, Maryland, United States of America; 3 Department of Molecular and Cellular Physiology, Stanford University School of Medicine, Stanford, California, United States of America; Stanford University School of Medicine, United States of America

## Abstract

Many signaling proteins including G protein-coupled receptors localize to primary cilia, regulating cellular processes including differentiation, proliferation, organogenesis, and tumorigenesis. Bardet-Biedl Syndrome (BBS) proteins are involved in maintaining ciliary function by mediating protein trafficking to the cilia. However, the mechanisms governing ciliary trafficking by BBS proteins are not well understood. Here, we show that a novel protein, Leucine-zipper transcription factor-like 1 (LZTFL1), interacts with a BBS protein complex known as the BBSome and regulates ciliary trafficking of this complex. We also show that all BBSome subunits and BBS3 (also known as ARL6) are required for BBSome ciliary entry and that reduction of LZTFL1 restores BBSome trafficking to cilia in BBS3 and BBS5 depleted cells. Finally, we found that BBS proteins and LZTFL1 regulate ciliary trafficking of hedgehog signal transducer, Smoothened. Our findings suggest that LZTFL1 is an important regulator of BBSome ciliary trafficking and hedgehog signaling.

## Author Summary

Primary cilia are considered to be a signaling hub coordinating multiple signaling pathways. Impairment of ciliary function results in developmental defects in vertebrates and also underlies many human disorders including obesity, polycystic kidney disease, and retinopathy. BBS is a prototypical human genetic disorder associated with ciliary dysfunction. Among the known BBS proteins, seven form a complex, the BBSome, which was recently defined as a coat complex transporting membrane proteins between plasma and ciliary membranes. However, the molecular mechanisms controlling BBSome trafficking and the cargos transported by the BBSome are not well understood. In this work, we performed tandem affinity purification using transgenic mice expressing one of the BBSome subunits and identified a novel protein, LZTFL1, as a BBSome interacting protein. We determined that LZTLF1 negatively regulates BBSome ciliary trafficking and that reduction of LZTFL1 activity can compensate for loss of certain BBS proteins and restores BBSome ciliary trafficking. Furthermore, we discovered that BBSome and LZTFL1 regulate ciliary trafficking of Smoothened, a 7-transmembrane Hedgehog signal transducer. Our findings identify an important player in cilia biology and provide novel insights into the regulation of Hedgehog signaling, a crucial signaling pathway for organizing the body plan, organogenesis, and tumorigenesis.

## Introduction

Primary cilia are microtubule-based subcellular organelles projecting from the surface of cells. Studies during the last decade have shown that primary cilia play essential roles in regulating cell cycle, embryonic development, and tissue homeostasis by acting as a cellular antenna transducing extracellular signals into the cells [1], [2]. Loss of cilia or ciliary dysfunction has been linked to a series of related genetic disorders in humans [3], [4]. These disorders, collectively termed ciliopathies, share common features such as cystic kidney disease, retinal degeneration, and polydactyly.

Bardet-Biedl Syndrome (BBS) is one of the human genetic disorders associated with ciliary dysfunction. Patients with BBS display obesity, polydactyly, retinal degeneration, renal abnormalities, diabetes, hypertension, hypogenitalism, and cognitive impairment. To date, as many as 16 genes have been reported to be involved in BBS [5], [6], [7], [8] (and references therein) and molecular functions of BBS proteins have begun to emerge. Among the known BBS proteins, seven proteins (BBS1, BBS2, BBS4, BBS5, BBS7, BBS8, BBS9) and BBIP10 form a stable complex, the BBSome, which mediates protein trafficking to the ciliary membrane [9], [10], [11]. BBS3 is a member of the Ras superfamily of small GTPases and controls BBSome recruitment to the membrane and BBSome ciliary entry [11]. Of the remaining, three BBS proteins (BBS6, BBS10, BBS12) form another complex with the CCT/TRiC family of group II chaperonins and mediate BBSome assembly [8].

Many receptor proteins and signaling molecules localize to cilia, and the BBSome is involved in transporting at least some of these proteins. For example, several G-protein coupled receptors such as MCHR1, SSTR3, and Dopamine receptor 1 (D1) fail to localize to or abnormally accumulate within the neuronal cilia in *Bbs2* and *Bbs4* null brains [12], [13]. In *Chlamydomonas bbs4* mutants, several proteins aberrantly accumulate within flagella [14]. However, most of the BBSome cargos are currently unknown in mammalian cells. Also unknown is how the trafficking activity of the BBSome is regulated.

In an effort to understand how BBSome function is regulated, we initiated studies to identify BBSome interacting proteins *in vivo*. In this work, we show that LZTFL1 interacts with the BBSome and negatively regulates its trafficking activity to the cilia. We also provide evidence that the BBSome and LZTFL1 are part of the transport mechanism of Sonic Hedgehog (SHH) signal transducer, Smoothened (SMO), that localizes to cilia [15].

## Results

### Transgenic LAP-BBS4 is functionally equivalent to endogenous Bbs4

To isolate BBSome interacting proteins *in vivo*, we generated a transgenic mouse line expressing *LAP-BBS4*, which allows localization studies and tandem affinity purification using GFP and S tags, under the control of cytomegalovirus (CMV) immediate early promoter. We used mouse testis, where BBS genes are the most abundantly expressed. Expression of *LAP-BBS4* in the testis is approximately 2–3 fold higher than that of endogenous *Bbs4* (Figure S1A). We first tested whether the recombinant LAP-BBS4 protein is functionally equivalent to endogenous Bbs4. We tested this by three criteria: 1) whether LAP-BBS4 physically associates with other BBSome subunits and is incorporated into the BBSome, 2) whether LAP-BBS4 properly localizes and reproduces the endogenous Bbs4 localization pattern, and 3) whether LAP-BBS4 can functionally rescue the BBS phenotype caused by loss of Bbs4. To confirm the incorporation of LAP-BBS4 into the BBSome, extracts from wild-type, *Bbs4^−/−^*, and *LAP-BBS4* transgenic mouse testes were subjected to co-immunoprecipitation (co-IP) with anti-GFP antibody. As shown in Figure S1B, pull-down of LAP-BBS4 efficiently co-precipitated all BBSome subunits tested (BBS1, BBS2 and BBS7). In spermatozoa, endogenous Bbs4 is found in the middle piece and the principle piece of the flagella (Figure S1C). Within the principle piece, Bbs4 staining is more intense at the proximal end and gradually decreases toward the distal end of the flagellum. LAP-BBS4 detected by GFP antibody shows a similar localization pattern. Finally, introduction of the *LAP-BBS4* transgene into *Bbs4^−/−^* mice restores sperm flagella, which are lost in *Bbs4^−/−^* mice (Figure S1D), and fertility to *Bbs4^−/−^* mice; all four transgenic *Bbs4^−/−^* males mated with wild-type females produced pups, while *Bbs4^−/−^* males without the transgene did not produce any pups. Based on these criteria, we concluded that LAP-BBS4 is functionally equivalent to endogenous Bbs4.

### Identification of Lztfl1 as a BBSome-interacting protein

**Figure 1:**
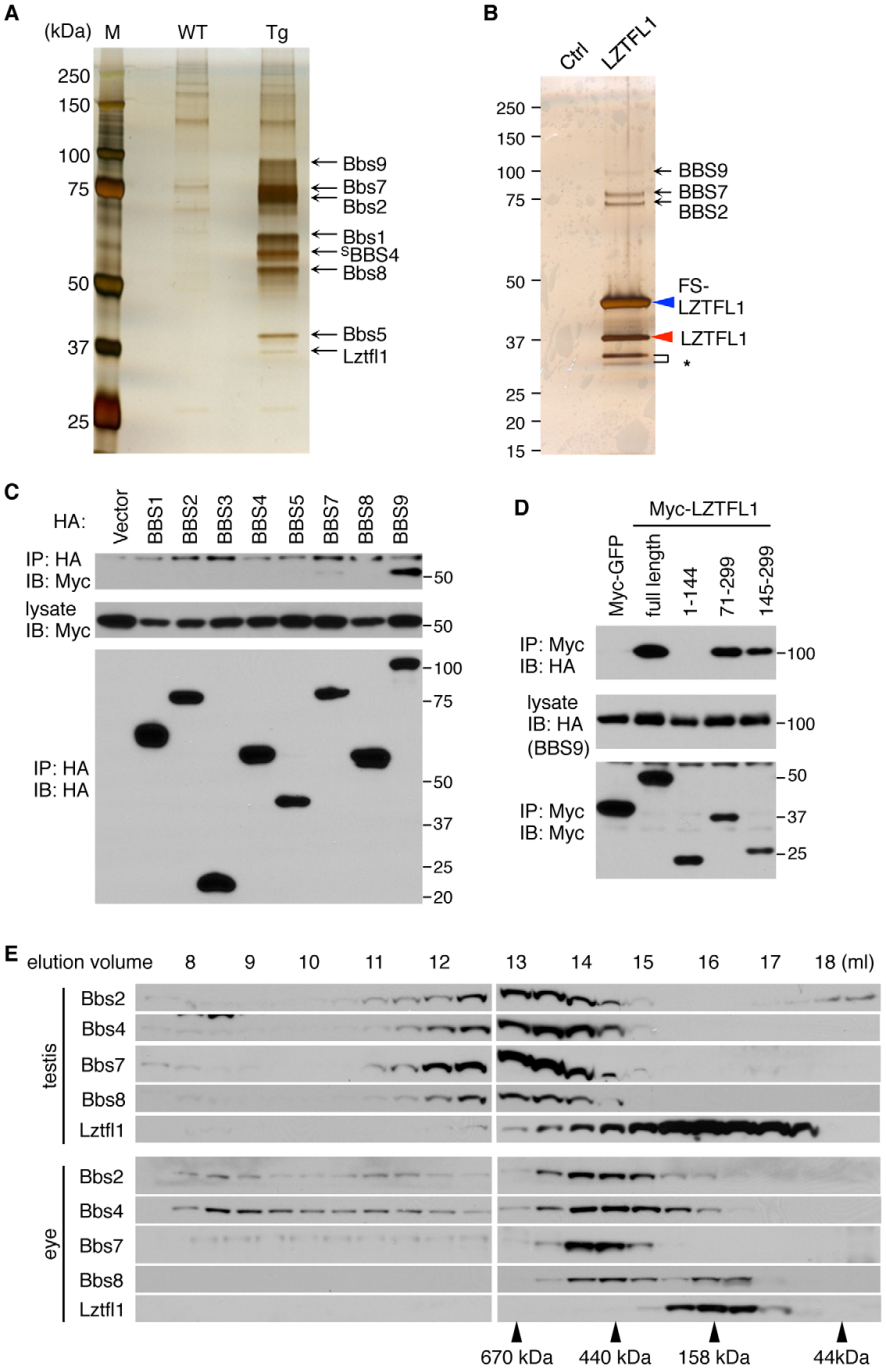
Identification of LZTFL1 as a BBSome interacting protein. (A) Co-purification of Lztfl1 as a BBSome-interacting protein from LAP-BBS4 transgenic mouse testis. Lysates from WT and Tg testes were subjected to TAP and purified proteins were separated by SDS-PAGE and silver-stained. Protein size markers (M) were loaded in the left lane. (B) FS-LZTFL1 (blue arrowhead) and its associated proteins were isolated by TAP from HEK293T cells and visualized by silver staining. Parental cells were used as a control. Red arrowhead indicates endogenous LZTFL1, and asterisk, two smaller forms of LZTFL1. (C) LZTFL1 interacts with BBS9 within the BBSome. Each subunit of the BBSome (HA-tagged) was co-transfected with Myc-tagged LZTFL1 into HEK293T cells and lysates were analyzed by co-immunoprecipitation (IP) using anti-HA antibodies. Bottom panel shows immunoprecipitated BBS proteins and middle, Myc-LZTFL1 in the lysates. The top panel shows Myc-LZTFL1 precipitated by anti-HA antibodies. (D) BBS9 binds to the C-terminal half of LZTFL1. Full-length and several deletion mutant variants of Myc-LZTFL1 were co-transfected with HA-BBS9 in HEK293T cells. Myc-GFP was used as a negative control and anti-Myc antibody was used for IP. Numbers indicate expressed portions of LZTFL1 in amino acid positions. (E) The vast majority of Lztfl1 is not associated with the BBSome. Lysates from WT mouse testis and eye were subjected to size exclusion chromatography using Superose-6 10/300 GL column. Eluted fractions were analyzed by SDS-PAGE followed by immunoblotting against indicated antibodies. Elution volume of protein standards is shown at the bottom.

Protein extracts from wild-type and *LAP-BBS4* transgenic testes were subjected to tandem affinity purification. In *LAP-BBS4* transgenic testis, all BBSome subunits (Bbs1, Bbs2, Bbs5, Bbs7, Bbs8, and Bbs9) were co-purified with LAP-BBS4, while no BBS proteins were purified when wild-type testes were used (Figure 1A and Table S1). In the LAP-BBS4 transgenic sample, one prominent additional protein was co-purified with BBSome subunits. Mass spectrometry analysis revealed that this protein is Leucine zipper transcription factor-like 1 (Lztfl1). To confirm the interaction and to identify other LZTFL1 interacting proteins, we generated a stable cell line expressing LZTFL1 with FLAG and S tags (FS-LZTFL1) and conducted tandem affinity purification. In this experiment, at least three BBSome subunits (BBS2, BBS7, and BBS9) were co-purified with FS-LZTFL1 (Figure 1B and Table S2). In addition, we found endogenous LZTFL1 proteins were co-purified, indicating that LZTFL1 forms homo-oligomers. Homo-oligomerization of LZTFL1 was confirmed by co-IP and *in vitro* crosslinking experiments (Figure S2D and S2E). We also found two additional, smaller isoforms of LZTFL1. Although it is unclear whether these smaller forms are *bona fide* LZTFL1 isoforms or cleavage products derived from the over-expressed FS-LZTFL1, EST database searches revealed the presence of smaller isoforms of LZTFL1 (AK093705 and AK303416) with predicted molecular weights approximately the same as the isoforms observed by us.

To determine the LZTFL1-interacting subunit of the BBSome, each individual subunit of the BBSome was co-transfected with LZTFL1 and analyzed for co-IP (Figure 1C). In this experiment, we found that BBS9 is the LZTFL1-interacting subunit of the BBSome. Based on interaction domain mapping studies, the C-terminal half of LZTFL1 (amino acid (aa) 145–299) was found to interact with BBS9 (Figure 1D). Within BBS9, the fragment containing aa 685–765 interacted with LZTFL1 (Figure S2F), which is a part of the α-helix domain at the C-terminus after the α/β platform domain [11]. In size exclusion chromatography, the peak of LZTFL1 was separated from that of the BBSome, suggesting that LZTFL1 is not a constitutive component of the BBSome and only a subset of LZTFL1 is associated with the BBSome (Figure 1E).

### LZTFL1 is a cytoplasmic protein without enrichment in cilia or basal bodies

*LZTFL1* maps to human chromosome 3p21.3, which is often deleted in several types of cancer [16]. Recently, tumor suppressor function of LZTFL1 has been proposed [17]. However, very little is known about the molecular functions of LZTFL1. To gain insight into the structure and functions of LZTFL1, we performed homology searches. Reciprocal BLAST searches yielded LZTFL1 orthologs in all vertebrates and the flagellate *Chlamydomonas reinhardtii,* but not in plants, amoebae, or fungi (Figure S2). LZTFL1 homologs are also not found in *Caenorhabditis elegans, Drosophila melanogaster*, and planaria (*Schmidtea mediterranea*). Sequence and secondary structure analyses indicated that LZTFL1 is mostly alpha-helical and has a coiled-coil domain in its C-terminal half (Figure S2). A leucine-zipper domain is present as part of the coiled-coil domain. InterPro Domain Scan analysis indicates that aa 212–295 of LZTFL1 has sequence homology to the t-SNARE domain (IPR010989). The structure of this domain in rat Syntaxin-1A (Stx1A) was previously determined (Figure S2C) [18]. The t-SNARE domain of Stx1A forms a rod-like α-helix and is involved in hetero-tetramer formation with other SNARE proteins. Purified Stx1A t-SNARE domain also forms a homo-tetramer [19], suggesting that LZTFL1 may form a similar structure.

**Figure 2:**
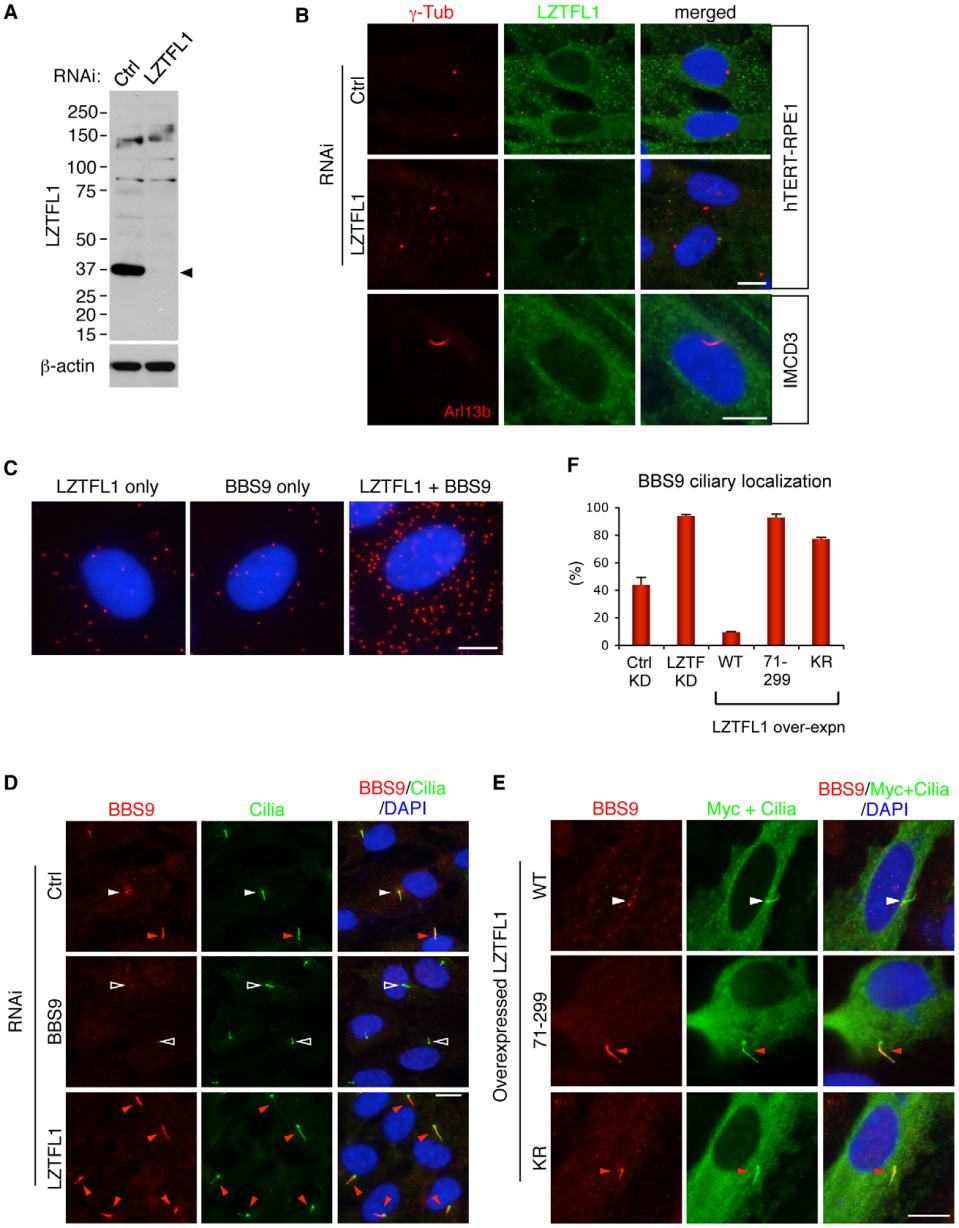
LZTFL1 regulates ciliary trafficking of the BBSome. (A) Verification of the specificity of anti-LZTFL1 antibody and siRNA-mediated knock-down of *LZTFL1* expression in hTERT-RPE1 cells. Arrowhead depicts the LZTFL1 protein band. β-actin was used as a loading control. (B) LZTFL1 localizes to the cytoplasm but not cilia or basal body. Localization of LZTFL1 (green) was probed with anti-LZTFL1 antibody in hTERT-RPE1 (top and middle rows) and IMCD3 (bottom row) cells after 30 hrs of serum withdrawal. Antibodies against γ-tubulin and Arl13b (red) were used to mark the basal body and cilia, respectively. Nuclei were stained with 4',6-diamidino-2-phenylindole (DAPI, blue). (C) LZTFL1-bound BBSome localizes to the cytoplasm. Left and middle panels are negative controls (background), where only either LZTFL1 or BBS9 antibodies was used. In the right panel, both LZTFL1 and BBS9 antibodies were used. Red dots (“blobs”) represent the protein complexes containing both LZTFL1 and BBS9 detected by *in situ* Proximity-mediated Ligation Assay (PLA) in hTERT-RPE1 cells. (D) BBS9 ciliary localization increases in LZTFL1 depleted cells. In ciliated RPE1 cells, BBS9 (red) shows two distinct localization patterns: ciliary (red arrowhead) and peri-centriolar (white arrowhead). Open arrowhead depicts the lack of BBS9 in *BBS9* siRNA transfected cells. Cilia (green) were marked by antibodies against acetylated tubulin and γ-tubulin. (E) Over-expression of LZTFL1 inhibits ciliary entry of BBS9. Myc-tagged LZTFL1 variants were transfected into RPE1 cells and BBS9 localization (red) was probed. Transfected cells were determined by using anti-Myc antibody (green). Scale bars, 10 µm. (F) Quantitation of BBS9 ciliary localization. The number of ciliated cells with ciliary BBS9 staining was counted. Results of knock-down (KD) experiments are average of four independent experiments with at least 100 cells counted in each experiment. Over-expression results are the average of two independent experiments with at least 40 cells counted in each experiment. Data are shown as means ± SEM.

We examined expression and localization of LZTFL1. Antibodies against LZTFL1 selectively recognized a protein band at ∼36 kDa in SDS-PAGE (Figure 2A and Figure S3B). This protein was diminished in cells transfected with siRNAs against LZTFL1, verifying the specificity of the antibody. *Lztfl1* expression was detected in almost every tissue tested except for skeletal muscle and white adipose tissue (Figure S3A). In immunolocalization studies using hTERT-RPE1 cells, LZTFL1 was detected throughout the cytoplasm (Figure 2B). In contrast to BBS proteins, which show ciliary and centriolar satellite localization (Figure 2D and Figure S4), we did not find enrichment of LZTFL1 around the centrosome or within cilia. GFP-tagged LZTFL1 also showed cytoplasmic localization with no enrichment in the cilia or centrosomes (data not shown). Similar results were obtained from IMCD3 cells and HEK293T cells (Figure 2B and Figure S3C). Consistent with this, we found most of the Lztfl1 immuno-reactivity in the inner segment of the photoreceptor cells and posterior side of the cell body of spermatozoa (Figure S3D, S3E). Since the localization pattern of LZTFL1 is significantly different from that of BBS proteins, we examined where the BBSome-LZTFL1 interaction occurs. Using the *in situ* proximity-mediated ligation assay [20], LZTFL1 bound BBSomes were found scattered throughout the cytoplasm (Figure 2C). These data indicate that LZTFL1 and BBSome interaction occurs within the cytoplasm.

### LZTFL1 is a specific regulator of BBSome ciliary trafficking

**Figure 3:**
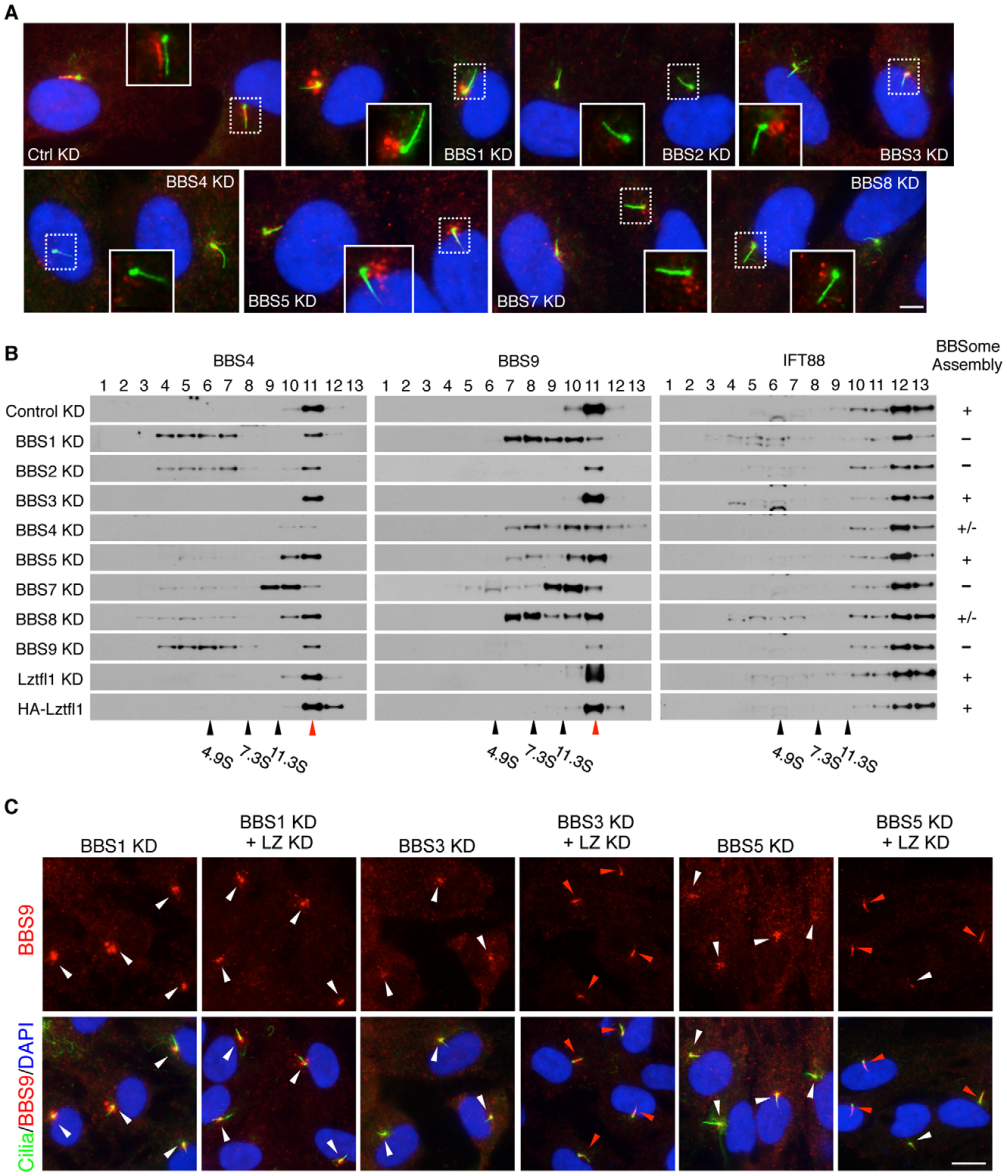
Reduction of LZTFL1 activity restores BBSome ciliary trafficking in BBS3 and BBS5 depleted cells. (A) All BBSome subunits are required for BBSome ciliary entry. RPE1 cells were transfected with siRNAs against each BBSome subunit and BBS3. Cilia and basal body are labeled with acetylated tubulin and γ-tubulin antibodies (green) and BBS9 is in red. The inlets are shifted overlay images of the boxed area. Scale bar, 5 µm. (B) BBSome status in the absence of individual BBSome components. Proteins from hTERT-RPE1 cells transfected with siRNAs against each BBS gene were separated by 10–40% sucrose gradient centrifugation and fractions were analyzed by immunoblotting using antibodies against BBS4, BBS9 and IFT88. Impact of LZTFL1 depletion and over-expression on BBSome assembly was also examined. Migration of molecular weight standards is shown at the bottom and red arrowhead indicates intact BBSome (14S). The BBSome assembly status was ranked by the amount of remaining intact BBSome (except for the depleted subunit) and summarized in the right. +: Normal BBSome assembly (more than 70% remaining), +/−: moderate decrease (30–70% remaining) severe decrease (less than 30% remaining). (C) Restoration of BBSome ciliary trafficking in BBS3 and BBS5 depleted cells by LZTFL1 depletion. RPE1 cells were transfected as indicated, and BBS9 (red) localization was examined. Scale bar, 10 µm.

Next, we sought to determine the biological functions of LZTFL1. Since the BBSome is involved in protein trafficking to the ciliary membrane and LZTFL1 interacts with the BBSome, we investigated whether LZTFL1 has any cilia related functions. To this end, we examined whether LZTFL1 is involved in cilia formation, cilia stability, or cilia length. We found no differences in cilia formation after serum withdrawal, cilia stability upon serum treatment, or the length of cilia in LZTFL1 depleted hTERT-RPE1 cells (data not shown). We next examined whether LZTFL1 is involved in BBSome assembly, BBS protein stability, or BBSome trafficking. We found no defects in BBSome assembly or BBS protein stability in LZTFL1 depleted cells (Figure 3B and data not shown). However, we noticed a dramatic alteration in BBSome localization when we ablated LZTFL1. Normally, BBS proteins localize either within cilia or around centrosomes (Figure 2D and Figure S4). In our experimental conditions, some 42% of control siRNA transfected cells show ciliary localization of BBS9 (Figure 2D, 2F). However, when LZTFL1 was depleted by RNA interference (RNAi), we observed a consistent and striking increase of BBS9 within the cilia with a concomitant decrease in the centriolar satellite pool of BBS9. In contrast, over-expression of wild-type LZTFL1 inhibited ciliary localization of BBS9 (Figure 2E, 2F). Interestingly, over-expression of an LZTFL1 deletion mutant, which lacks the N-terminal 70 amino acids, behaved as a dominant negative form and increased ciliary localization of BBS9. Substitution of two highly conserved basic amino acids (KR) within that region (Figure S2A) was sufficient to cause the same increase in BBS9 ciliary localization. Similar results were observed for BBS4 and BBS8 (Figure S4), indicating that LZTFL1 regulates ciliary localization of the entire BBSome rather than merely BBS9. Combined with the LZTFL1 localization and *in situ* PLA results, our data indicate that LZTFL1 binds to the BBSome in the cytoplasm and inhibits BBSome ciliary entry. Alternatively, LZTFL1 may promote ciliary exit of the BBSome. Our data also suggest that LZTFL1 has bipartite functional domains; the C-terminal half of LZTFL1 is responsible for BBSome binding and the N-terminal half is for regulating BBSome trafficking activity.

To test whether LZTFL1 regulates general intraflagellar transport (IFT), we examined localization of IFT proteins in LZTFL1 over-expressing and depleted cells. In contrast to BBS proteins, IFT57 and IFT88 were found in almost every cilium (Figures S4 and 5). Depletion of LZTFL1 did not further increase the frequency of ciliary localization of IFT proteins or the fluorescence intensity. More importantly, over-expression of wild-type or mutant variants of LZTFL1 had no impact on ciliary localization of IFT proteins. These data suggest that LZTFL1 is a specific regulator of BBSome ciliary trafficking but not general IFT.

### Requirement of all BBSome subunits for BBSome ciliary localization

Previously, several BBSome subunits have been shown to be essential for BBSome ciliary localization in *C. elegans* and *C. reinhardtii* [14], [21]. However, it has not been systematically investigated which BBSome subunits are essential for BBSome ciliary localization and which are dispensable. To address this, we transfected siRNAs against each BBSome subunit and BBS3, which is involved in BBSome ciliary trafficking [11], into hTERT-RPE1 cells and probed localization of BBS8 and BBS9. Quantitative real-time PCR results confirmed efficient knock-down of each BBS gene expression after siRNA transfection (Figure S6). By using immunofluorescence microscopy, we found that all BBSome subunits and BBS3 are required for BBSome ciliary localization and loss of any single BBSome subunit precluded ciliary entry of the BBSome (Figure 3A and Figure S7). Interestingly, however, the localization pattern of BBS9 (and BBS8) was distinct depending on which BBSome subunit was depleted, suggesting differences in their roles within the BBSome. For example, in BBS1 depleted cells, both BBS8 and BBS9 showed concentric enrichment around the centrosomes with a great increase in the staining intensity (Figure 3A and Figure S7A). This suggests that BBSome components may form aggregates near the centrosomes in the absence of BBS1. Alternatively, BBS1 may be required to return the BBSome back to the cytoplasm. Sucrose gradient ultracentrifugation results are more consistent with the second possibility (Figure 3B). In BBS2 depleted cells, overall staining intensity of BBS9 was greatly decreased. When we measured BBS9 protein level in BBS2 depleted cells, the amount of BBS9 was significantly reduced compared to control siRNA or other BBS gene siRNA transfected cells (Figure S6B). In addition, we found BBS2 protein level was also significantly decreased in BBS9 depleted cells, suggesting that BBS2 and BBS9 are dependent on each other for stable expression. Together, these data indicate that only the intact BBSome can enter the cilia.

To gain insight into the molecular basis of this requirement of each BBSome subunit for BBSome ciliary entry, we investigated the status of BBSome assembly after individual BBSome subunit depletion (Figure 3B). To this end, hTERT-PRE1 cells were transfected with siRNAs against control and each BBS gene, and cell lysates were analyzed by 10–40% sucrose gradient ultracentrifugation. Consistent with the previous result and formation of the BBSome [9], BBS4 and BBS9 were found in the same fraction in control siRNA transfected cells. However, in BBS1 depleted cells, BBS4 and BBS9 were found in separate and lower molecular weight fractions, indicating that BBS4 and BBS9 were not associated in the absence of BBS1. Similarly, depletion of BBS2 and BBS9 also caused disintegration of the BBSome with a significant reduction in BBS9 levels. In BBS4 and BBS8 depleted cells, although there was some disassembly of the BBSome, a significant proportion of the BBSome was still found to be intact. In BBS7 depleted cells, BBS4 and BBS9 were found in the same fractions but the peak was significantly shifted from that of control cells, suggesting that at least one additional subunit is missing from the BBSome in the absence of BBS7. Of note are BBS3 and BBS5 depleted cells. In these cells, the vast majority of the BBSome remained intact, suggesting that BBS3 and BBS5 are not required for BBSome assembly. LZTFL1 depletion or over-expression did not cause any change in BBSome assembly, suggesting that LZTFL1 does not function by modulating BBSome assembly. IFT88, a component of the IFT-B subcomplex, was found in separate fractions from the BBSome and none of the BBS or LZTFL1 perturbations caused any change in IFT88 migration. Together, these data suggest that BBS1, BBS2, BBS7, and BBS9, all of which have β-propeller domains, are required for BBSome assembly (e.g. forming a core scaffold and required for recruitment of at least one additional BBSome subunit), while BBS4, BBS5, and BBS8 have relatively minor or no impact on BBSome assembly and are likely to be in the periphery of the BBSome.

### Depletion of LZTFL1 restores BBSome ciliary localization in BBS3 and BBS5 depleted cells

Since LZTFL1 is a negative regulator of BBSome ciliary entry, we tested whether LZTFL1 depletion can rescue the BBSome mislocalization phenotype caused by loss of BBSome subunits. We ablated *LZTFL1* expression together with each of the BBSome subunits by RNAi and probed localization of BBS8 and BBS9. Indeed, LZTFL1 knock-down significantly increased ciliary localization of BBS8 in most cells, particularly in BBS3 and BBS5 depleted cells (Figure S7). Ciliary localization of BBS9 was also rescued by LZTFL1 knock-down in all cases except for BBS1 and BBS4 depleted cells (Figure 3C and Figure S7). It is unclear whether the more efficient rescue of BBS9 ciliary localization compared to BBS8 is due to the higher sensitivity of anti-BBS9 antibody or whether it represents features of partial BBSome complexes lacking some BBSome subunits (such as BBS8). Whichever is the case, it is clear that LZTFL1 knock-down can restore ciliary localization of the BBSome at least in BBS3 and BBS5 depleted cells. It is noteworthy that BBS3 and BBS5 are the BBS proteins that have minimal impact on BBSome assembly. Therefore, as long as the BBSome forms, reducing LZTFL1 activity can restore BBSome trafficking to cilia.

### BBSome and LZTFL1 regulate SMO ciliary trafficking

**Figure 4:**
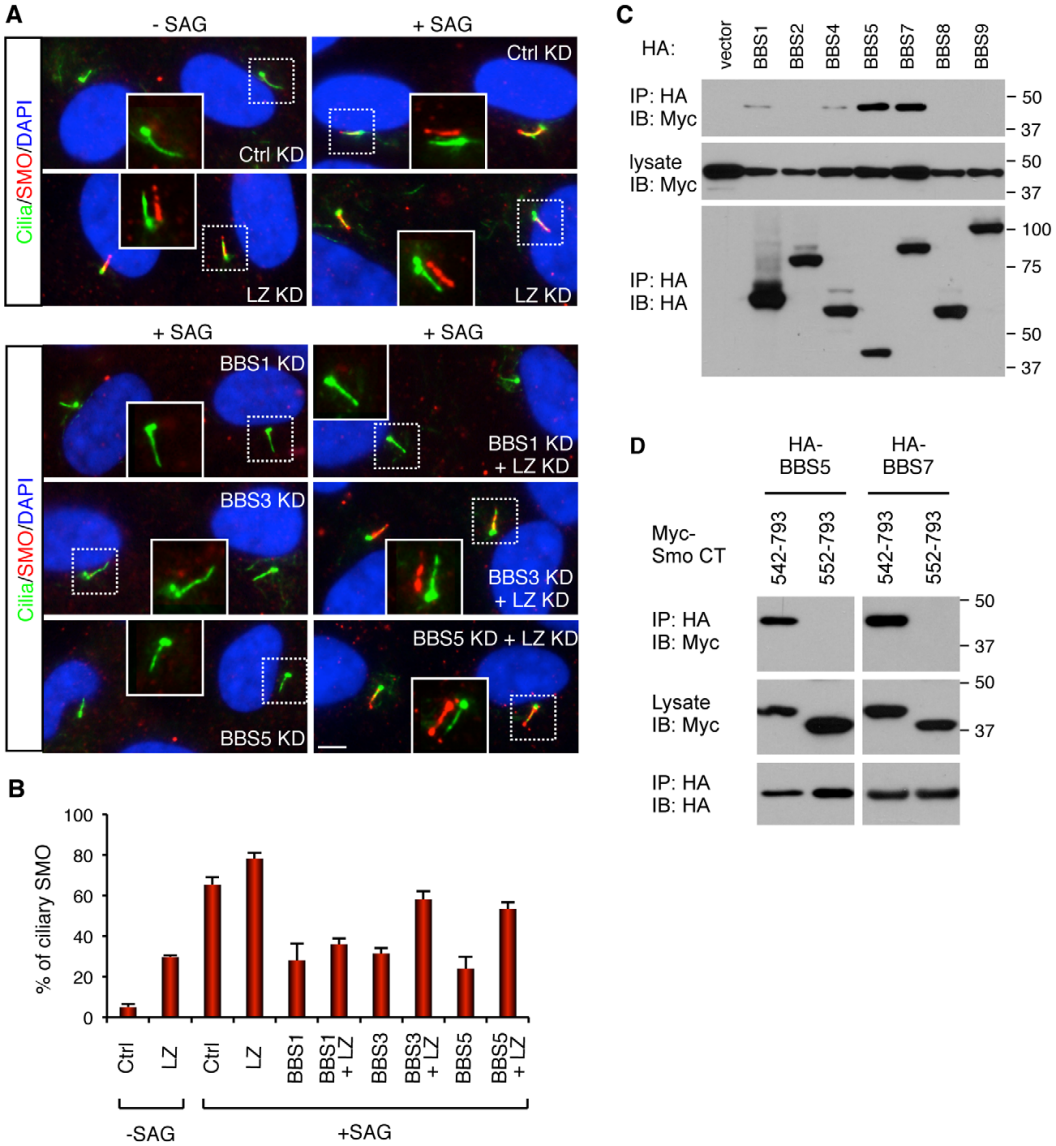
BBSome and LZTFL1 regulate SMO ciliary trafficking. (A) BBSome is required for SMO ciliary localization, while LZTFL1 inhibits it. hTERT-RPE1 cells were depleted with BBSome subunits and LZTFL1 (LZ) by RNAi. After 24 hrs of serum withdrawal, cells were incubated with or without SMO agonist (SAG) for 4 hrs. Cells were labeled with antibodies to acetylated tubulin and γ-tubulin (green) and SMO (red). Scale bar, 5 µm. (B) Quantitation of SMO ciliary localization. Graphs are average percentages of SMO positive cilia from four independent experiments with minimum 90 cells counted in each experiment. Data are shown as means ± SEM. (C) Physical interaction between BBSome and Smo C-terminal cytoplasmic tail domain. Indicated BBSome subunits (HA-tagged) were co-transfected with Myc-tagged Smo cytoplasmic tail domain (aa 542–793) and lysates were subjected to co-IP using anti-HA antibodies. (D) Deletion of 10 amino acids, which contains the WR motif, from the N-terminus of Smo cytoplasmic tail domain abolishes the interaction between Smo and BBS proteins.

Since polydactyly is one of the cardinal features of BBS and a hallmark phenotype of Sonic Hedgehog (SHH) signaling defect, we examined roles of BBSome and LZTFL1 in the SHH pathway. We first examined the requirement of LZTFL1 for SMO ciliary localization in hTERT-RPE1 cells, which express SMO endogenously. In control siRNA transfected cells, SMO localizes to the cilia in response to SMO agonist (SAG) but not in SAG-untreated cells (Figure 4). However, in LZTFL1 depleted cells, ciliary localization of SMO was found in a significant number of cells even without SAG treatment and further increased by SAG treatment. For BBS genes, we tested BBS1, BBS3, and BBS5: two genes (BBS3 and BSB5) that LZTFL1 depletion can restore ciliary trafficking of the BBSome, and one gene (BBS1) that cannot be restored with LZTFL1 depletion. In contrast to LZTFL1 depleted cells, ciliary localization of SMO was significantly decreased in BBS depleted, SAG-treated cells. Consistent with the restoration of BBSome trafficking to cilia in LZTFL1 depleted cells, ablation of LZTFL1 expression in BBS3 and BBS5, but not in BBS1, depleted cells restored ciliary localization of SMO. These data indicate that BBSome function facilitates ciliary localization of SMO and that LZTFL1 suppresses SMO localization to cilia.

Although we were not able to detect physical interactions between the endogenous BBSome and SMO in hTERT-RPE1 cells, presumably due to the transient nature of the interaction and the difficulty of extracting membrane proteins without disrupting protein-protein interactions, we found that several BBS proteins can associate with the C-terminal cytoplasmic tail domain of SMO (aa 542–793) in transiently transfected cells (Figure 4C). Previously, two amino acids (WR; aa 549–550) immediately downstream of the 7th transmembrane domain of SMO were shown to be essential for SMO ciliary localization [15]. Deletion of this WR motif from the cytoplasmic tail of SMO abolished interaction between BBS proteins and SMO (Figure 4D). These data suggest that the BBSome may directly interact with SMO and mediates SMO ciliary localization.

Finally, we investigated whether downstream HH target gene expression is affected by loss of BBSome and LZTFL1 function. In hTERT-RPE1 cells, HH target gene *GLI1* expression was relatively mildly induced by SAG treatment (Figure S8A). Depletion of BBS1, BBS3, and BBS5 modestly but consistently reduced *GLI1* expression. We also used mouse embryonic fibroblast (MEF) cells, which show robust responsiveness (Figure S8B). Consistent with the results from hTERT-PRE1 cells, knock-down of BBS gene expression significantly reduced *Gli1* expression in MEF cells. Although statistically not significant compared with BBS3 and BBS5 single knockdowns, reduction of Lztfl1 activity tends to restore *Gli1* expression in Bbs3 and Bbs5 depleted cells. Interestingly, while LZTFL1 depletion resulted in ciliary translocation of SMO even in SAG-untreated cells, *GLI1* expression did not increase in LZTFL1 depleted cells, suggesting that SMO accumulated within the cilia in LZTFL1 depleted cells is not activated. This is consistent with the idea that Smo is constantly transported in and out of the cilia even in the inactive state [22], [23] and the recent finding of the 2-step model of SMO activation [24]. Finally, we used MEF cells derived from our *Bbs2* and *Bbs4* null embryos [25], [26] and found similar reductions in *Gli1* expression upon SAG treatment (Figure S8C). Together, our data suggest that the BBSome and LZTFL1 are a part of the Smo ciliary trafficking mechanisms and contribute to the cellular responsiveness to the SHH signaling agonist.

## Discussion

BBSome functions as a coat complex to transport membrane proteins between plasma and ciliary membranes [11]. In this work, we identify LZTFL1 as a negative regulator of BBSome trafficking to the ciliary membrane. Our data indicate that LZTFL1 associates with the BBSome within the cytoplasm and inhibits ciliary entry of the BBSome. Alternatively, LZTFL1 may promote the exit of BBSomes from the cilia. Although we cannot completely rule out the second possibility, currently available data favor the entry inhibition model over the exit promotion model. First, LZTFL1 does not show any enrichment around the basal body or within the cilia either by immunological methods or by GFP-fused recombinant protein. Many proteins involved in vesicle trafficking commonly show some degree of enrichment around the donor compartment. BBSomes also show enrichment around the basal body and within the cilia with a gradual increase toward the ciliary base, which is consistent with the model that the BBSome is recruited to the plasma membrane near the basal body for ciliary entry and to the ciliary membrane at the ciliary base for exit. However, LZTFL1 does not show this localization pattern. Second, although BBSomes show enrichment around the basal body and within the cilia, LZTFL1-associated BBSomes are found scattered throughout the cytoplasm, which is consistent with the idea that LZTFL1 associates with the BBSome within the cytoplasm and limits ciliary access of the BBSome. These observations strongly favor the BBSome ciliary entry inhibition model. Localization of LZTFL1 to the cytoplasm with cilia-related function is similar to the recently characterized seahorse/Lrrc6, which also localizes to the cytoplasm and regulates cilia-mediated processes [27].

Our findings indicate that every BBS gene tested so far is required for ciliary entry of the BBSome and loss of any single BBS protein commonly results in a failure of BBSome ciliary trafficking. This is not limited to BBSome subunits but also found with depletion of other BBS proteins that are not part of the BBSome including BBS3, BBS6, BBS10, and BBS12 (this study and S.S. and V.C.S. unpublished results). These findings suggest that a failure of BBSome ciliary trafficking is a common cellular feature of BBS and support the idea that BBS results from a trafficking defect to the cilia membrane. However, the precise mechanism leading to BBSome mis-localization is different depending on the missing BBS proteins. For example, BBS1, BBS2, BBS7, and BBS9, which commonly contain β-propeller domains, are likely to form the scaffold/core of the BBSome and required to recruit other BBSome subunits. BBS6, BBS10, and BBS12 were previously shown to be required for BBSome assembly by interacting with these β-propeller domain containing BBS proteins [8]. BBS4, BBS5, and BBS8 are likely to be at the periphery of the BBSome and have limited impact on BBSome assembly. However, BBS4 interacts with p150^glued^ subunit of the cytoplasmic dynein machinery and may link the BBSome to the cytoplasmic dynein motor protein [28]. BBS5 was shown to interact with PIPs and may be involved in the association of the BBSome to the membrane [9]. The function of BBS8 is currently unknown and requires further characterization. Despite these functional differences, all BBS proteins are essential for BBSome assembly or ciliary trafficking and only the holo-BBSome enters the cilia. BBSome mis-localization may be used as a cell-based assay to evaluate BBS candidate genes. Remarkably, reducing LZTFL1 activity restores BBSome trafficking to the cilia at least in BBS3 and BBS5 depleted cells. It appears that decreased LZTFL1 activity can compensate for the loss of certain BBS proteins, which are required for BBSome ciliary entry, as long as the BBSome is formed. This implies that certain BBS subtypes may be treated by modulating LZTFL1 activities.

Polydactyly is one of the cardinal features of BBS found in the vast majority of human BBS patients [3] and also a hallmark phenotype of disrupted SHH signaling or IFT function [2], [29], [30]. Several additional features including mid-facial defects and neural crest cell migration defects found in BBS mutant animals are also linked to SHH signaling defects [31]. Therefore, it is speculated that BBS proteins may be involved in SHH signaling. In this work, we show that BBS proteins are involved in ciliary trafficking of SMO. We found that the SMO cytoplasmic tail domain physical interacts with the BBSome at least in overexpressed conditions, while ciliary localization defective mutant SMO does not. Furthermore, ablation of LZTFL1 increases SMO ciliary localization. These data indicate that BBS proteins and LZTFL1 are at least part of the SMO ciliary trafficking mechanism. It remains to be shown that ciliary transport of Smo occurs together with the BBSome (e.g. by live cell imaging) and in a directly BBSome-dependent manner.

Our data also suggest the presence of alternative ciliary trafficking mechanisms for SMO. For example, although ciliary localization of SMO is significantly reduced by BBSome depletion, it is not completely abolished, while ciliary localization of BBS8 and BBS9 is severely reduced. HH target gene *GLI1* expression is also relatively mildly affected by BBS protein depletion. This suggests that the BBSome is only part of the transport mechanism and that there is likely to be another mechanism by which SMO is transported to the cilia. Currently, it is unknown whether IFT proteins can directly mediate SMO ciliary trafficking or are involved indirectly. These findings suggest that although BBS proteins are involved in Smo ciliary trafficking, the presence of alternative mechanisms is sufficient to support normal development of neural tubes and survival in BBS mutants. In addition, while polydactyly is very common in human BBS patients, none of the BBS mouse models generated so far display polydactyly as is seen in Smo or Shh mutants [2], implying a potential species-specific requirement of BBS proteins or differential threshold in SHH signaling in the limb bud.

While BBS and IFT proteins are part of the transport machinery (like a train) to deliver cargos to and from the cilia, NPHP, MKS, and Tectonic proteins localize to the transition zone and function like a station (or immigration official) to control the entry and exit of the ciliary proteins. For example, Garcia-Gonzalo, *et al*. recently showed that the Tectonic complex, which consists of Tctn1, Tctn2, Tctn3, Mks1, Mks2, Mks3, Cc2d2a, B9d1, and Cep290, localizes to the transition zone of cilia and controls ciliary membrane composition [32]. NPHP1, NPHP4, and NPHP8 form another complex at the transition zone and functions as a ‘gate keeper’ together with other NPHP and MKS proteins [33], [34], [35]. These NPHP, MKS, and Tctn protein functions also appear to be partly redundant. In this model, one can envisage certain ciliary proteins transported by the BBSome and allowed to enter the cilia by the Tectonic complex. Some other proteins may be transported by a non-BBSomal mechanism and granted access to cilia by the Tectonic complex or by NPHP1–4–8 complex. LZTFL1 is a specific regulator of BBSome ciliary trafficking activity. Some other proteins may regulate activities of IFT complex or specific transition zone complexes. Obtaining a complete picture of ciliary protein transport will be the focus of future studies to understand the precise mechanisms of cilia-related disorders.

## Materials and Methods

### Ethics statement

All animal work in this study was approved by the University Animal Care and Use Committee at the University of Iowa.

### Antibodies, plasmids, and reagents

Expression vectors for BBS genes were published [8]. Human and mouse LZTFL1 cDNA clones (NM_020347 and NM_033322) were purchased from OpenBiosystems and subcloned into CS2 plasmids with Myc, FLAG, HA, or FS (FLAG and S) tag after PCR amplification. Site-directed mutagenesis was performed by using QuikChange protocol (Agilent) and PfuUltra II Fusion HS DNA polymerase (Agilent). Small interfering RNAs (siRNAs) were purchased from Dharmacon (ON-TARGETplus SMARTpool) and transfected at 100 nM concentration for *Gli1* gene expression analysis and at 50 nM concentration for all other experiments with RNAiMAX (Invitrogen) following manufacturer's protocol.

Antibodies against BBS1, BBS2, BBS4 and BBS7 were described previously [9]. To produce rabbit polyclonal antibody for mouse Lztfl1, recombinant NusA-Lztfl1 protein (full-length) was purified using HIS-SELECT Nickel Affinity Gel (Sigma) and used as antigen to immunize rabbits (Proteintech Group). Smo-N antibody was a generous gift from Dr. R. Rohatgi (Stanford University). Other antibodies used were purchased from the following sources: mouse monoclonal antibodies against acetylated tubulin (6–11B-1; Sigma), γ-tubulin (GTU-88; Sigma), LZTFL1 (7F6; Abnova), Myc (9E10; SantaCruz), FLAG (M2; Sigma), HA (F-7; SantaCruz), β-actin (AC-15; Sigma), rabbit polyclonal antibodies against ARL13B (Proteintech Group), BBS7 (Proteintech Group), BBS8 (Sigma), BBS9 (Sigma), γ-tubulin (Sigma), IFT57 (Proteintech Group), IFT88 (Proteintech Group), Smo (Abcam), and rabbit monoclonal antibody against GFP (Invitrogen).

### Transgenic mouse and Hematoxylin/Eosin (H&E) staining

The LAP-BBS4 transgenic mouse line was generated by injecting the LAP-BBS4 expression cassette of the pLAP-BBS4 construct [9] into 1-cell pronuclear stage mouse embryo from B6SJL (C57BL/6J X SJL/J; Jackson Laboratory) strain in the University of Iowa Transgenic Animal Facility. Transgenic animals were maintained in the mixed background of C57BL/6J and 129/SvJ. Genotype was determined by PCR using the following primers (5′-GTCCTGCTGGAGTTCGTGAC-3′ and 5′-GGCGAAATATCAATGCTTGG-3′). Progenies from three founders expressed the *LAP-BBS4* transgene. In two lines, LAP-BBS4 levels were less than 50% of endogenous Bbs4 in the testis and only one line expressed LAP-BBS4 higher than endogenous Bbs4 (Figure S1). This line was used for the entire study. Bbs4 knock-out mouse model and hematoxylin/eosin staining was previously described [26].

### Tandem affinity purification (TAP) and co-immunoprecipitation (Co-IP)

Testes from six wild-type and LAP-BBS4 transgenic animals were used for TAP. Proteins were extracted with lysis buffer (50 mM HEPES pH 7.0, 200 mM KCl, 1% Triton X-100, 1 mM EGTA, 1 mM MgCl_2_, 0.5 mM DTT, 10% glycerol) supplemented with Complete Protease Inhibitor cocktail (Roche Applied Science). The remaining TAP procedure was described previously [9]. TAP of FS-LZTFL1 was conducted with HEK293T cells stably expressing FS-LZTFL1 or parental cells. Cell lysates from twenty 15-cm dishes were loaded onto anti-FLAG affinity gel (M2; Sigma), and bound proteins were eluted with 3xFLAG peptide (100 µg/mL; Sigma). Eluate was loaded onto S-protein affinity gel (Novagen), and bound proteins were eluted in 2x SDS-PAGE sample loading buffer. Purified proteins were separated in 4–12% NuPAGE gels (Invitrogen) and visualized with SilverQuest Silver Staining Kit (Invitrogen). Excised gel slices were submitted to the University of Iowa Proteomics Facility and protein identities were determined by mass spectrometry using LTQ XL linear ion trap mass spectrometer (Thermo Scientific). Co-IP was performed as previously described [8].

### Sucrose gradient ultracentrifugation and size exclusion chromatography

Protein extract from one 10-cm dish of hTERT-RPE1 cell was concentrated with Microcon Centrifugal Filter Devices (50,000 MWCO; Millipore), loaded on a 4 ml 10–40% sucrose gradient in PBST (138 mM NaCl, 2.7 mM KCl, 8 mM Na_2_HPO_4_, 1.5 mM KH_2_PO_4_, 0.04% Triton X-100), and spun at 166,400 x G_avg_ for 13 hrs. Fractions (∼210 µl) were collected from the bottom using a 26 G needle and concentrated by TCA/acetone precipitation. Proteins were re-suspended in equal volume of 2x SDS-PAGE sample loading buffer and analyzed by SDS-PAGE and immunoblotting. Size exclusion chromatography was previously described [8]. Briefly, protein extracts from testis and eye were concentrated by Amicon Ultra-15 (30 kDa; Millipore) and loaded on a Superose-6 10/300 GL column (GE Healthcare). Eluted fractions were TCA/acetone precipitated and re-suspended in 2x SDS loading buffer. The column was calibrated with Gel Filtration Standard (Bio-Rad).

### Quantitative real-time PCR, immunofluorescence microscopy, and *in situ* proximity-mediated ligation assay (PLA)

hTERT-RPE1 cells were maintained in DMEM/F12 media (Invitrogen) supplemented with 10% FBS. Immortalized MEF cells expressing Smo-YFP was kindly provided by Dr. M. Scott (Stanford University) and cultured in DMEM with 10% FBS. Primary MEF cells from wild-type and *Bbs2* and *Bbs4* null embryos [25], [26] were prepared following a standard protocol at embryonic day 13.5. Cells were transfected with siRNAs using RNAiMAX for 48 hrs and further incubated in serum-free medium for 24 hrs for ciliation. For RNA extraction, cells were treated with 100 nM SAG (EMD Chemicals) for additional 18 hrs in a fresh serum-free medium. Total RNA was extracted using TRIzol Reagent (Invitrogen) following manufacturer's instruction. Quantitative PCR was performed as previously described [8]. RPL19 mRNA levels were used for normalization and ΔΔCt method [36] was used to calculate fold inductions. The PCR products were confirmed by melt-curve analysis and sequencing. Knock-down efficiencies of BBS genes were measured by qPCR and samples with more than 90% reduction in BBS gene expression levels were used for *GLI1* gene expression analysis. PCR primer sequences are in Table S3. For immunofluorescence microscopy, cells were seeded on glass coverslips in 24-well plates and transfected with siRNAs using RNAiMAX or with plasmid DNAs using FuGENE HD (Roche Applied Science). Cells were cultured for 72 hrs before fixation with the last 30 hrs in serum-free medium to induce ciliogenesis. For SAG treatment, 100 nM SAG was added to fresh serum-free medium and incubated for 4 hrs at 37°C. Cells were fixed with cold methanol, blocked with 5% BSA and 2% normal goat serum in PBST, and incubated with primary antibodies in the blocking buffer. Primary antibodies were visualized by Alexa Fluor 488 goat anti-mouse IgG (Invitrogen) and Alexa Fluor 568 goat anti-rabbit IgG (Invitrogen). Coverslips were mounted on VectaShield mounting medium with DAPI (Vector Lab), and images were taken with Olympus IX71 microscope. For *in situ* PLA, Duolink *in situ* PLA kit with anti-mouse PLUS probe and anti-rabbit MINUS probe (OLINK Bioscience) was used with mouse monoclonal antibody for LZTFL1 (Abnova) and rabbit polyclonal antibody for BBS9 (Sigma) following manufacturer's instruction. Briefly, RPE1 cells were seeded onto an 8-well Lab-Tek II chamber slide (Nunc) and treated as for immunofluorescence microscopy until the primary antibody binding step. After washing, cells were decorated with PLA PLUS and MINUS probes (1∶20 dilution) for 2 hrs in a 37°C humidified chamber. Hybridization and ligation of probes, amplification, and final SSC washing were performed per manufacturer's instruction in a humidified chamber. Complex formation was detected by Duolink Detection kit 563 (OLINK Bioscience) and Olympus IX71 microscope.

### Homology search, secondary structure prediction, and modeling

Homology search, secondary structure prediction, and modeling were performed by using human LZTFL1 protein sequence (NP_065080), SWISS-MODEL Workspace, and InterProScan [37], [38]. Rat Stx1A H3 domain (PDB: 1hvv) was used as a template and aligned with human LZTFL1 aa 212–284.

### Protein cross-linking

For paraformaldehyde (PFA) cross-linking, HEK293T cells were incubated with 1% PFA in DMEM at 37°C for 10 min. For cross-linking with photoreactive amino acids, cells were cultured in Dulbecco's Modified Eagle's Limiting Medium (DMEM-LM; Pierce) with L-Photo-Leucine and L-Photo-Methionine (Pierce) supplemented with 10% dialyzed FBS (Pierce). Cross-linking was performed following manufacturer's instruction. Cells were irradiated in the UV Stratalinker 2400 (Stratagene) 5-cm below the UV lamp for 6 minutes. After cross-linking, cells were washed with PBS three times and lysed in the lysis buffer (50 mM Tris pH 7.0, 150 mM NaCl, 0.5% Triton X-100, 0.5% CHAPS, 2 mM EDTA, 2 mM NaF, 2 mM NaVO4) supplemented with Complete Protease Inhibitor cocktail (Roche Applied Science). Protein extracts were loaded onto a 4–12% SDS-PAGE gel and subjected to immunoblotting.

### Immunofluorescence microscopy for the retinal sections and spermatozoa

Wild-type mice at 2–3 months of age were used for retinal sections. Animals were perfused with 4% PFA in PBS (2.5 ml/min, 50 ml), and excised eyes were post-fixed for 30 min in the same solution. After washing with PBS, eyes were frozen in OCT and sectioned using cryostat with CryoJane system (myNeuroLab) with 7 µm thickness. For mouse spermatozoa, testis and epididymis from wild-type animals were minced by forceps in Ham's F10 solution (Invitrogen) and incubated for 20 minutes at 37°C in 5% CO_2_ incubator. The turbid upper fraction (“swim-up” fraction) was collected using wide-opening tips and spread onto positively charged slide glasses. Excess liquid was slowly aspirated and samples were air-dried. After fixation with 4% PFA in PBS for 5 min, samples were processed for immunofluorescence following standard protocol.

## Supporting Information

Figure S1 Transgenic LAP-BBS4 is functionally equivalent to endogenous Bbs4. (A) Expression of the *LAP-BBS4* transgene in the testis. Lysates from wild-type (WT), LAP-BBS4 transgenic (Tg) testes from *Bbs4^−/+^, Bbs4^+/−^,* and *Bbs4^−/−^* animals were subjected to SDS-PAGE and immunoblotting with indicated antibodies. Closed arrowhead marks endogenous Bbs4 and open arrowhead, LAP-BBS4. Size markers are shown on the right. Bbs7 and β-actin were used as loading controls. (B) LAP-BBS4 physically associates with other BBSome subunits. Lysates from WT and Tg testes were subjected to immunoprecipitation (IP) with anti-GFP antibody and precipitated proteins were analyzed by immunoblotting with indicated antibodies. (C) LAP-BBS4 reproduces the localization pattern of endogenous Bbs4. Sperm cells from WT and Tg animals were stained with antibodies against acetylated α-tubulin (Tub), Bbs4, and GFP. No staining was found in the acrosomes with the GFP antibody, suggesting that the staining in this area with anti-Bbs4 antibody is likely to be from cross-reacting proteins. Scale bar, 10 µm. (D) Introduction of LAP-BBS4 to the *Bbs4^−/−^* (4KO) animals restores sperm flagella. Scale bar, 50 µm.
(PDF)

Figure S2 LZTFL1 structure and interaction. (A) Amino acid sequence alignment of LZTFL1 homologs from human (*Homo sapiens*), mouse (*Mus musculus*), chicken (*Gallus gallus*), frog (*Xenopus tropicalis*), zebrafish (*Danio rerio*), *Ciona intestinalis,* and *Chlamydomonas reinhardtii*. The blue line above the amino acid (aa) sequences represents the predicted coiled-coil domain following [16]. The red line above the aa sequences designates the t-SNARE domain determined by PSI-BLAST. Red boxes mark the basic amino acids mutagenized in the KR (to AS) construct (see Figure 2). Asterisks represent residues conserved in all species and colons, similar residues. (B,C) Prediction of LZTFL1 secondary structure (B) and modeling of C-terminal t-SNARE domain of LZTFL1 (C). Rat Stx1A H3 domain (PDB: 1hvv, chain A) was used as a template and aligned with human LZTFL1 aa 212–284. Lower panels are 90° rotated images of the upper panels. (D) Homo-oligomerization of LZTFL1. HA-LZTFL1 was co-transfected with Myc-GFP or Myc-LZTFL1 into HEK293T cells and IP was performed with anti-Myc antibody. (E) HEK293T cells were cross-linked with 1% paraformaldehyde (PFA) in PBS or with L-Photo-Leucine and L-Photo-Methionine (Photo-L/M). Lysates were analyzed by SDS-PAGE and immunoblotting. Monomeric (mono) and dimeric (di) forms of LZTFL1 were marked. Several forms of dimeric LZTFL1 were found. (F) LZTFL1-interaction domain mapping in BBS9. Deletion mutants of HA-BBS9 were co-transfected with FLAG-LZTFL1. ΔCC indicates deletion of the coiled-coil domain (amino acids 378–408).
(PDF)

Figure S3 LZTFL1 expression and localization. (A) Expression profile of Lztfl1 in mouse tissues. Equal amounts of proteins from indicated tissues were loaded on a SDS-PAGE gel and Lztfl1 protein levels were examined by immunoblotting. α-tubulin was used as a loading control. (B) Protein extracts from HEK293T cells transfected with siRNAs against control or *LZTFL1* gene were analyzed by immunoblotting. β-actin was used as a loading control. (C) Cytoplasmic localization of LZTFL1 in HEK293T cells. Cells were transfected with indicated siRNAs. In lower panels (LZTFL1 siRNA transfected), presumptive untransfected cells were included in the picture for direct comparison of LZTFL1 staining intensities within the picture. (D) Localization of Lztfl1 to the inner segment (IS) of photoreceptor cells. OS; outer segment, ONL; outer nuclear layer, INL; inner nuclear layer. Scale bar: 50 µm. (E) Localization of Lztfl1 in mouse spermatozoa. Scale bar: 10 µm.
(PDF)

Figure S4 LZTFL1 regulates BBSome localization to the cilia. (A) Gallery of BBS protein localization. Localization of LAP-BBS4, endogenous BBS8, BBS9, and IFT88 was examined in hTERT-RPE1 cells. Cilia and basal bodies are marked by acetylated tubulin and γ-tubulin (green) and BBS proteins and IFT88 are in red. BBS proteins are found either within the cilia (red arrowhead) or around the centrosomes (white arrowhead), with a concomitant decrease in the other compartment. In contrast, IFT88 is found within the cilia in virtually every cell. (B) Depletion of LZTFL1 increases ciliary localization of LAP-BBS4. hTERT-RPE1 cells were transfected with siRNAs as indicated. Cilia were labeled with anti-acetylated tubulin and anti-γ-tubulin antibodies (green) and LAP-BBS4, detected by anti-GFP antibody, is in red. (C) While over-expression of wild-type (WT) Myc-LZTFL1 suppresses ciliary localization of LAP-BBS4, N-terminal deletion mutant (Myc-LZTFL1 aa 71–299) increases ciliary LAP-BBS4. Transfected cells were determined by anti-Myc antibody (green). (D) LZTFL1 depletion increases ciliary localization of BBS8. Scale bars, 10 µm.
(PDF)

Figure S5 LZTFL1 does not regulate general IFT. hTERT-RPE1 cells were transfected with siRNAs (A) or Myc-LZTFL1 variants (B) and localization of IFT88 (red) was probed. Scale bar, 10 µm.
(PDF)

Figure S6 Suppression of BBS gene expression by RNAi. hTERT-RPE1 cells were transfected with siRNAs as indicated and relative mRNA levels (A) and protein levels (B) were compared by quantitative PCR and immunoblotting, respectively.
(PDF)

Figure S7 Reduction of LZTFL1 activity restores BBSome ciliary trafficking in BBS3 and BBS5 depleted cells. (A) Localization of BBS8 was probed by immunofluorescence after transfecting indicated siRNAs into hTERT-RPE1 cells. Cilia are labeled with antibodies for acetylated tubulin and γ-tubulin (green) and BBS8 is in red. (B) RPE1 cells were transfected with indicated BBS and LZTFL1 (Lz) siRNAs and BBS8 localization (red) was examined. (C) BBS9 localization (red) was examined after depleting BBS genes and LZTFL1. Note that some panels are also shown in Figure 6. Scale bar, 10 µm. (D,E) Summary of BBSome ciliary localization. Graphs represent average percentages of BBS8 (D) and BBS9 (E) positive cilia from at least two independent experiments with minimum 100 cells counted in each experiment. Error bars represent standard errors.
(PDF)

Figure S8 Expression of HH target gene *GLI1* upon BBS protein depletion. (A) Expression of *GLI1* in hTERT-RPE1 cells. RPE1 cells were transfected with siRNAs as indicated and treated with or without 100 nM SAG for 18 hrs. *GLI1* mRNA levels were measured by quantitative PCR. Data are shown as means ± SEM of two independent experiments. (B) Expression of *Gli1* in MEF cells. Immortalized MEF cells were transfected with siRNAs as indicated and SAG treatment was performed as in (A). Data are shown as means ± SEM of three independent experiments. Asterisks indicate statistically significant differences compared to control KD cells with SAG treatment (*t*-test; P<0.05). (C) Reductions in *Gli1* expression in *Bbs2^−/−^* and *Bbs4^−/−^* MEF cells upon SAG treatment. *Gli1* expressions in *Bbs2^−/−^* and *Bbs4^−/−^* MEF cells were compared with that of WT MEF cells from the same liter. Reverse transcription (RT) reactions without reverse transcriptase (-) were used as a negative control. Shown are representative results from a semi-quantitative PCR experiment.
(PDF)

Table S1 Summary of mass spectrometry analysis of the LAP-BBS4 eluate.
(DOCX)

Table S2 Summary of mass spectrometry analysis of the FS-LZTFL1 eluate.
(DOCX)

Table S3 Quantitative real-time PCR primer sequences.
(DOCX)
