# Supplementary figures and images for: A Novel Protein LZTFL1 Regulates Ciliary Trafficking of the BBSome and Smoothened
Source: PLoS Genet. 2011 Nov 3;7(11):e1002358. doi: 10.1371/journal.pgen.1002358 (PMC3207910; doi:10.1371/journal.pgen.1002358)

Figure S1

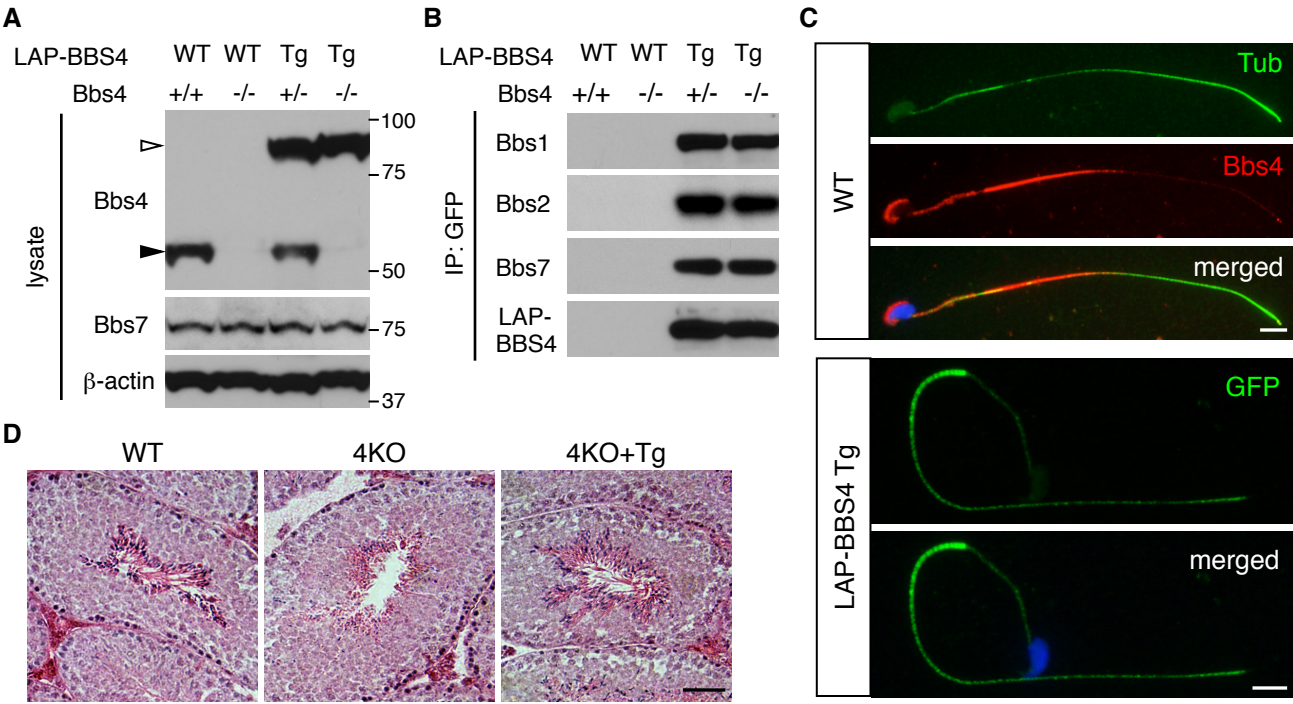

Supplement: Figure S1 — Transgenic LAP-BBS4 is functionally equivalent to endogenous Bbs4. (A) Expression of the LAP-BBS4 transgene in the testis. Lysates from wild-type (WT), LAP-BBS4 transgenic (Tg) testes from Bbs4−/+, Bbs4+/−, and Bbs4−/− animals were subjected to SDS-PAGE and immunoblotting with indicated antibodies. Closed arrowhead marks endogenous Bbs4 and open arrowhead, LAP-BBS4. Size markers are shown on the right. Bbs7 and β-actin were used as loading controls. (B) LAP-BBS4 physically associates with other BBSome subunits. Lysates from WT and Tg testes were subjected to immunoprecipitation (IP) with anti-GFP antibody and precipitated proteins were analyzed by immunoblotting with indicated antibodies. (C) LAP-BBS4 reproduces the localization pattern of endogenous Bbs4. Sperm cells from WT and Tg animals were stained with antibodies against acetylated α-tubulin (Tub), Bbs4, and GFP. No staining was found in the acrosomes with the GFP antibody, suggesting that the staining in this area with anti-Bbs4 antibody is likely to be from cross-reacting proteins. Scale bar, 10 µm. (D) Introduction of LAP-BBS4 to the Bbs4−/− (4KO) animals restores sperm flagella. Scale bar, 50 µm. (PDF) [file pgen.1002358.s001.pdf]

Figure S3

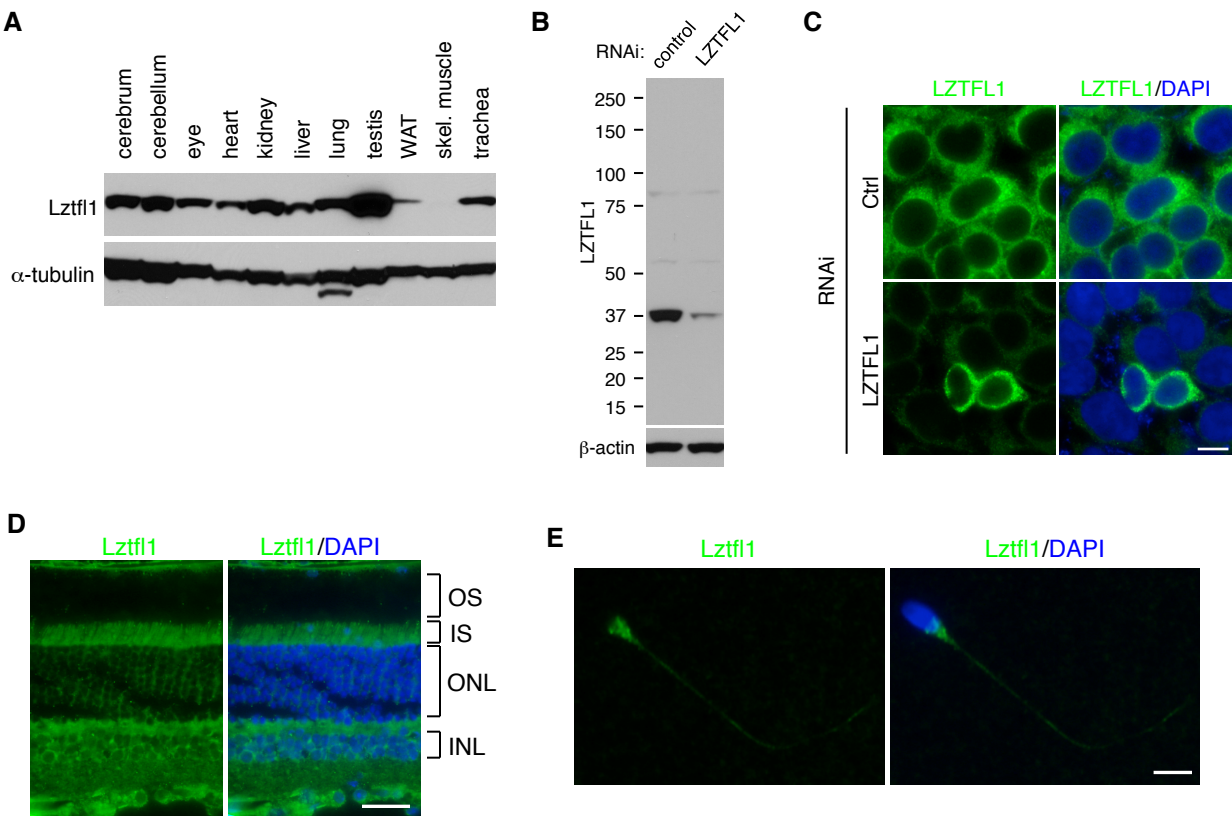

Supplement: Figure S3 — LZTFL1 expression and localization. (A) Expression profile of Lztfl1 in mouse tissues. Equal amounts of proteins from indicated tissues were loaded on a SDS-PAGE gel and Lztfl1 protein levels were examined by immunoblotting. α-tubulin was used as a loading control. (B) Protein extracts from HEK293T cells transfected with siRNAs against control or LZTFL1 gene were analyzed by immunoblotting. β-actin was used as a loading control. (C) Cytoplasmic localization of LZTFL1 in HEK293T cells. Cells were transfected with indicated siRNAs. In lower panels (LZTFL1 siRNA transfected), presumptive untransfected cells were included in the picture for direct comparison of LZTFL1 staining intensities within the picture. (D) Localization of Lztfl1 to the inner segment (IS) of photoreceptor cells. OS; outer segment, ONL; outer nuclear layer, INL; inner nuclear layer. Scale bar: 50 µm. (E) Localization of Lztfl1 in mouse spermatozoa. Scale bar: 10 µm. (PDF) [file pgen.1002358.s003.pdf]

Figure S4

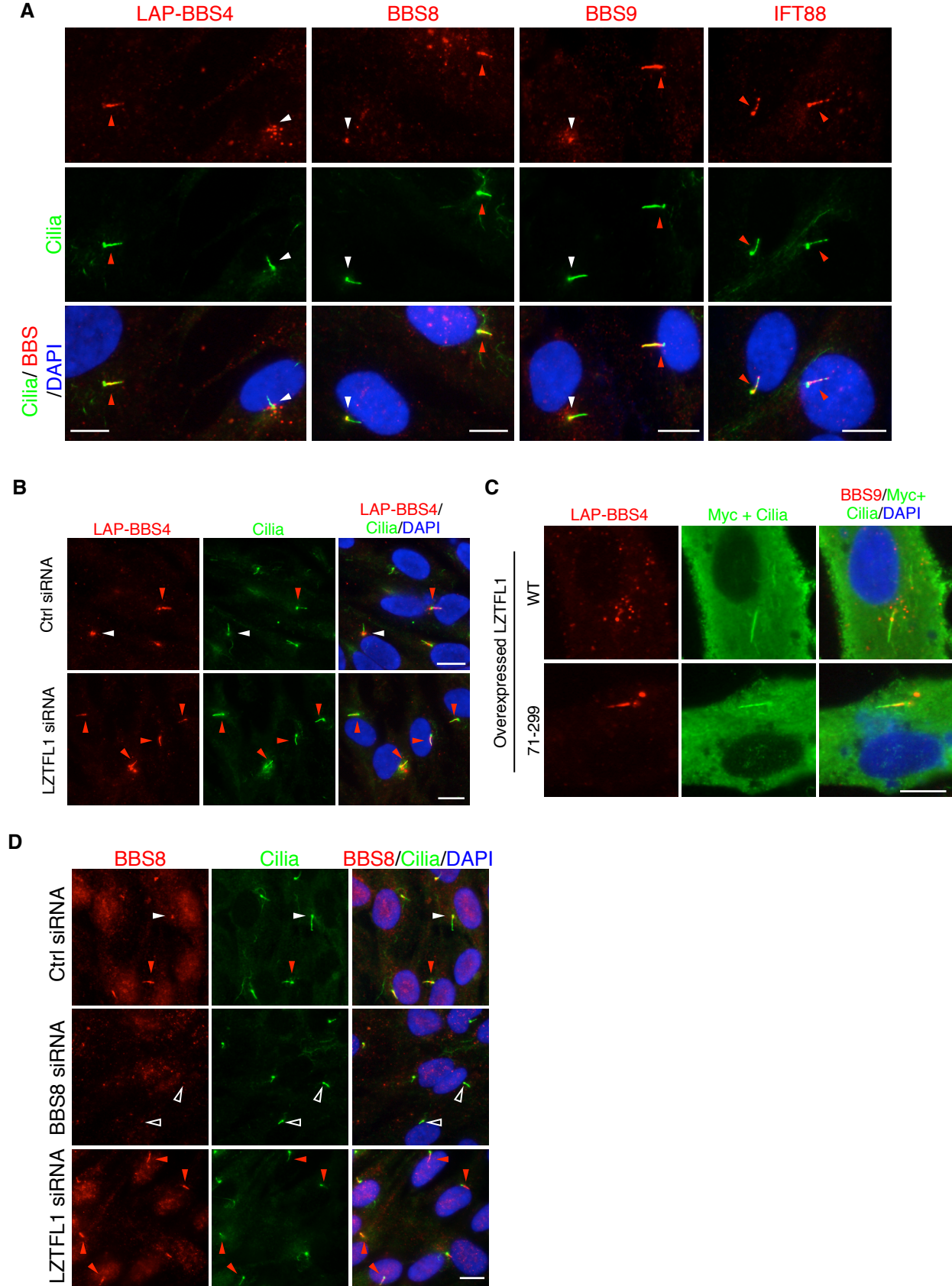

Supplement: Figure S4 — LZTFL1 regulates BBSome localization to the cilia. (A) Gallery of BBS protein localization. Localization of LAP-BBS4, endogenous BBS8, BBS9, and IFT88 was examined in hTERT-RPE1 cells. Cilia and basal bodies are marked by acetylated tubulin and γ-tubulin (green) and BBS proteins and IFT88 are in red. BBS proteins are found either within the cilia (red arrowhead) or around the centrosomes (white arrowhead), with a concomitant decrease in the other compartment. In contrast, IFT88 is found within the cilia in virtually every cell. (B) Depletion of LZTFL1 increases ciliary localization of LAP-BBS4. hTERT-RPE1 cells were transfected with siRNAs as indicated. Cilia were labeled with anti-acetylated tubulin and anti-γ-tubulin antibodies (green) and LAP-BBS4, detected by anti-GFP antibody, is in red. (C) While over-expression of wild-type (WT) Myc-LZTFL1 suppresses ciliary localization of LAP-BBS4, N-terminal deletion mutant (Myc-LZTFL1 aa 71–299) increases ciliary LAP-BBS4. Transfected cells were determined by anti-Myc antibody (green). (D) LZTFL1 depletion increases ciliary localization of BBS8. Scale bars, 10 µm. (PDF) [file pgen.1002358.s004.pdf]

Figure S5

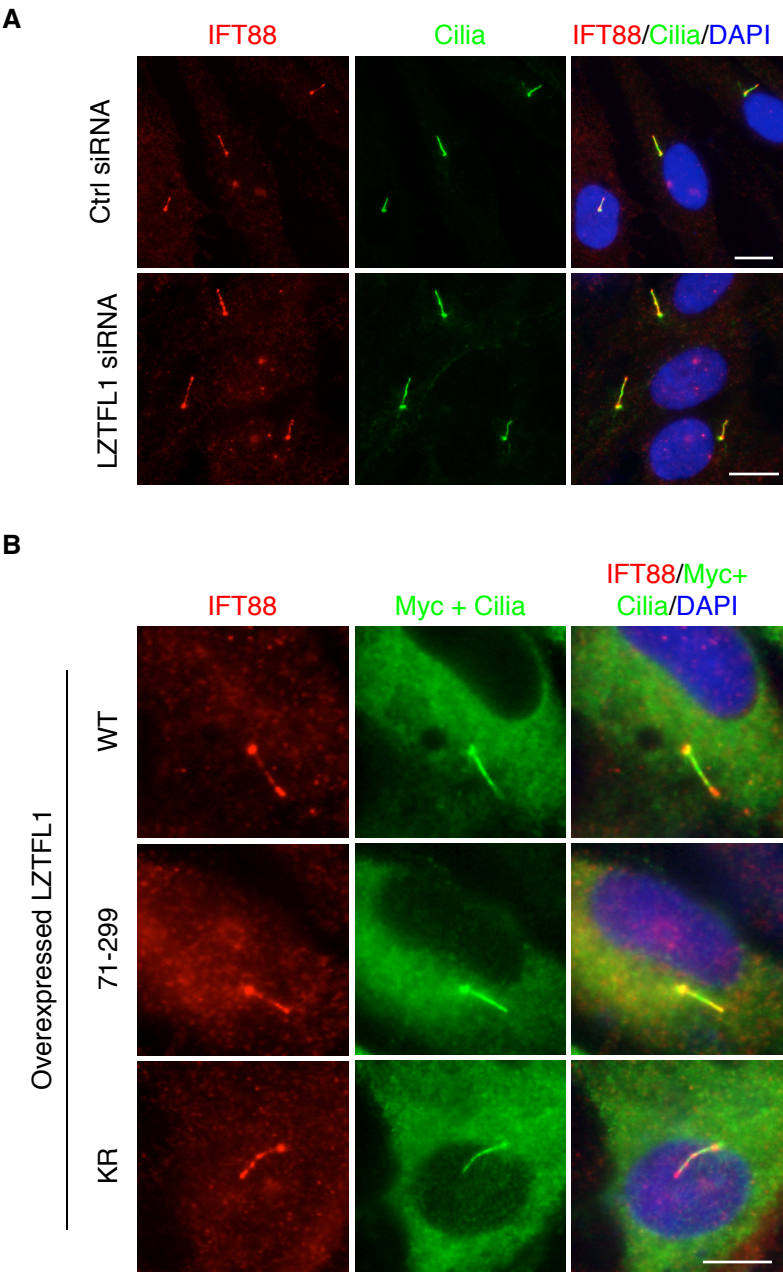

Supplement: Figure S5 — LZTFL1 does not regulate general IFT. hTERT-RPE1 cells were transfected with siRNAs (A) or Myc-LZTFL1 variants (B) and localization of IFT88 (red) was probed. Scale bar, 10 µm. (PDF) [file pgen.1002358.s005.pdf]

**Figure S6**

**A**

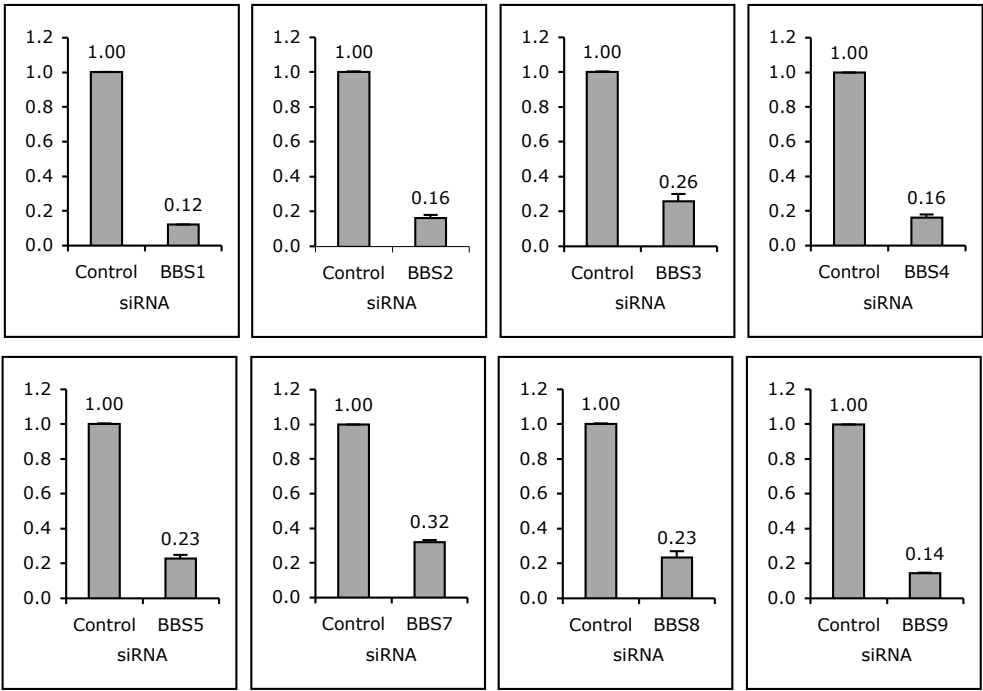

**B**

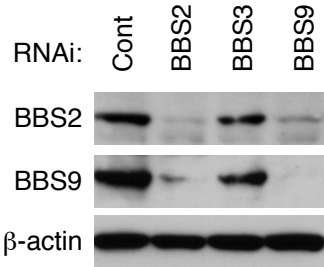

Supplement: Figure S6 — Suppression of BBS gene expression by RNAi. hTERT-RPE1 cells were transfected with siRNAs as indicated and relative mRNA levels (A) and protein levels (B) were compared by quantitative PCR and immunoblotting, respectively. (PDF) [file pgen.1002358.s006.pdf]

Figure S7

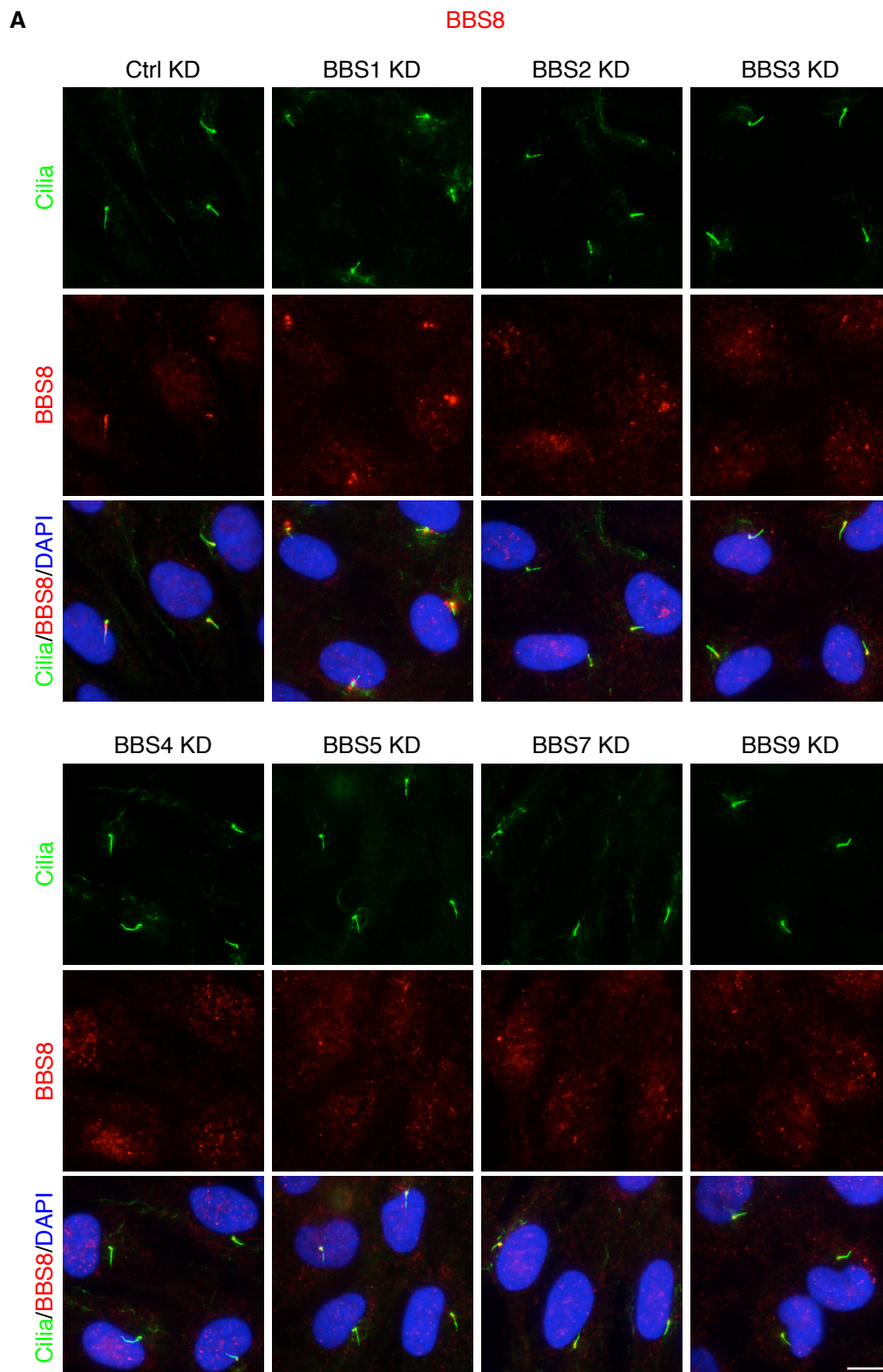

**B**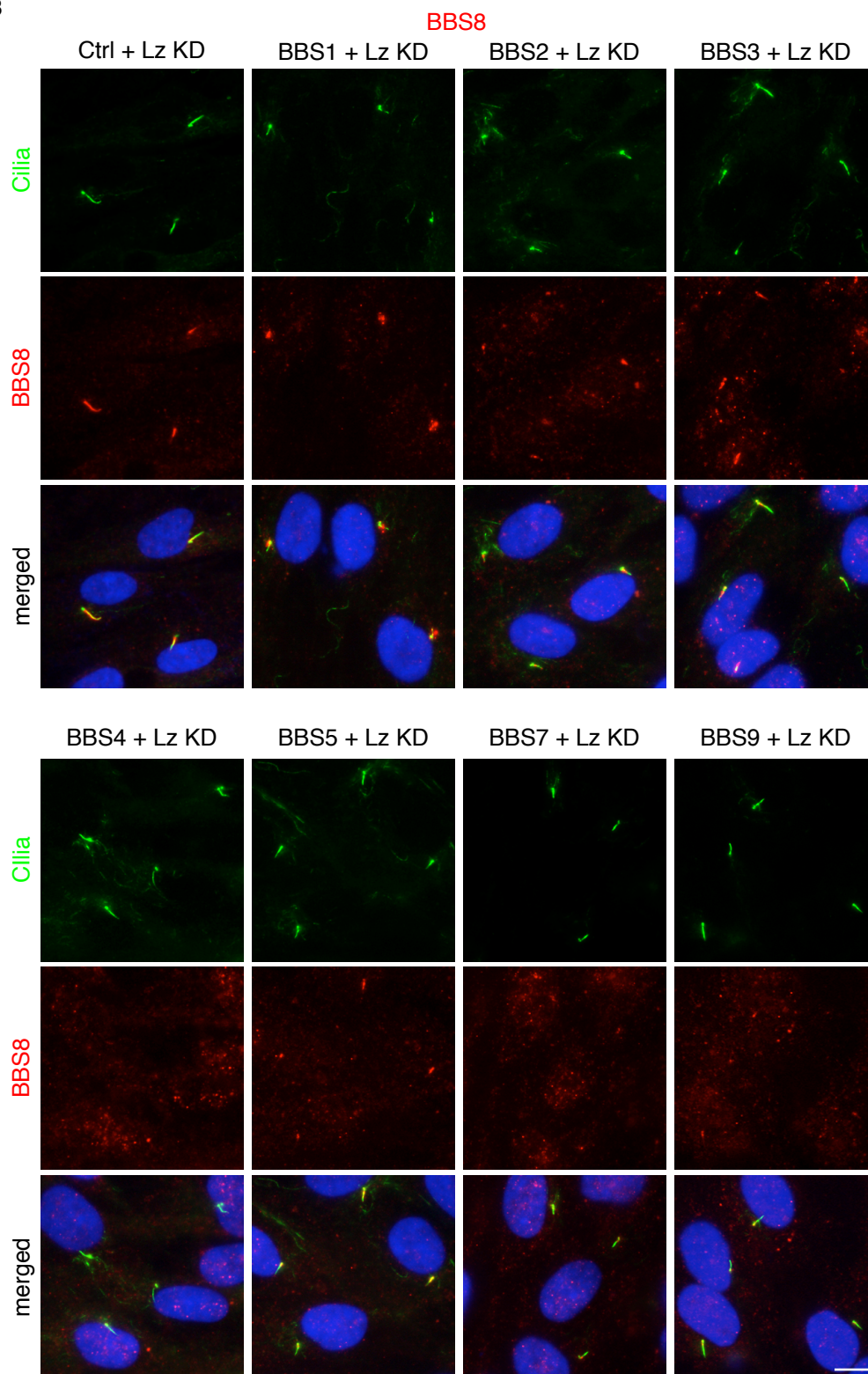

**C**

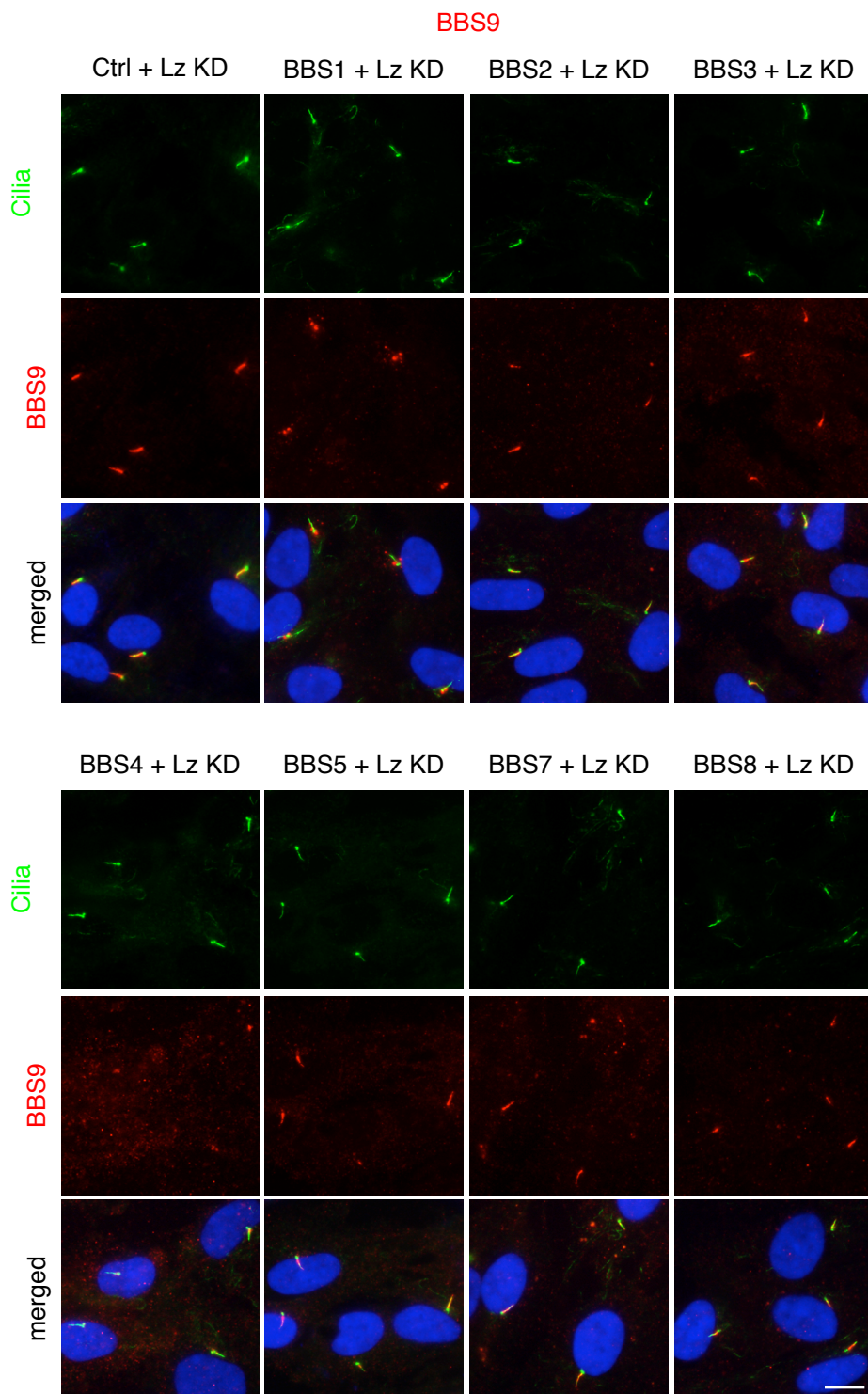

**D**

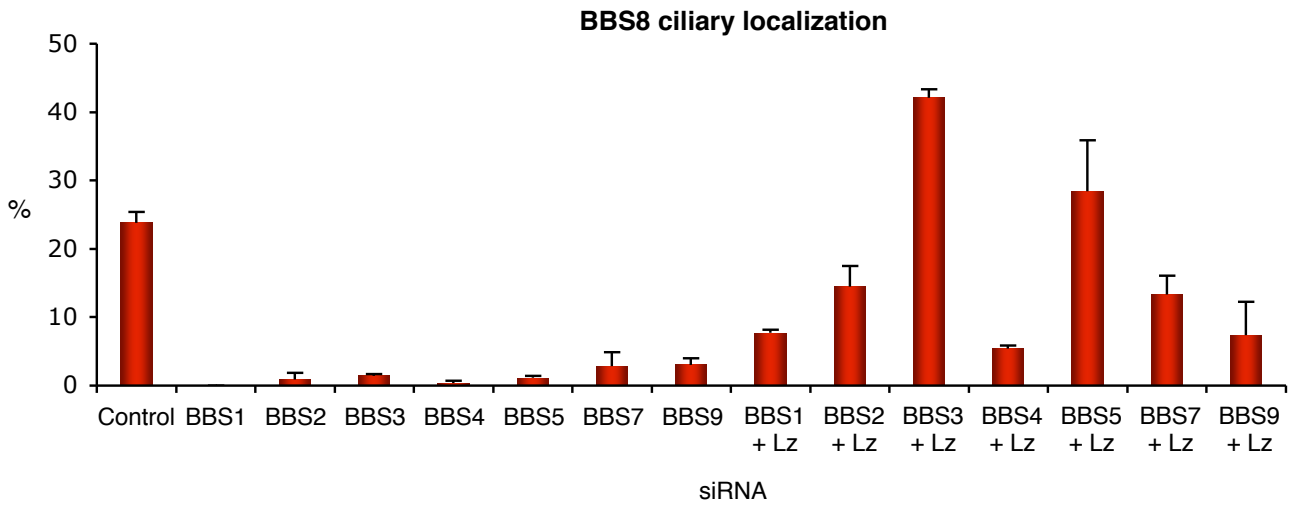

**E**

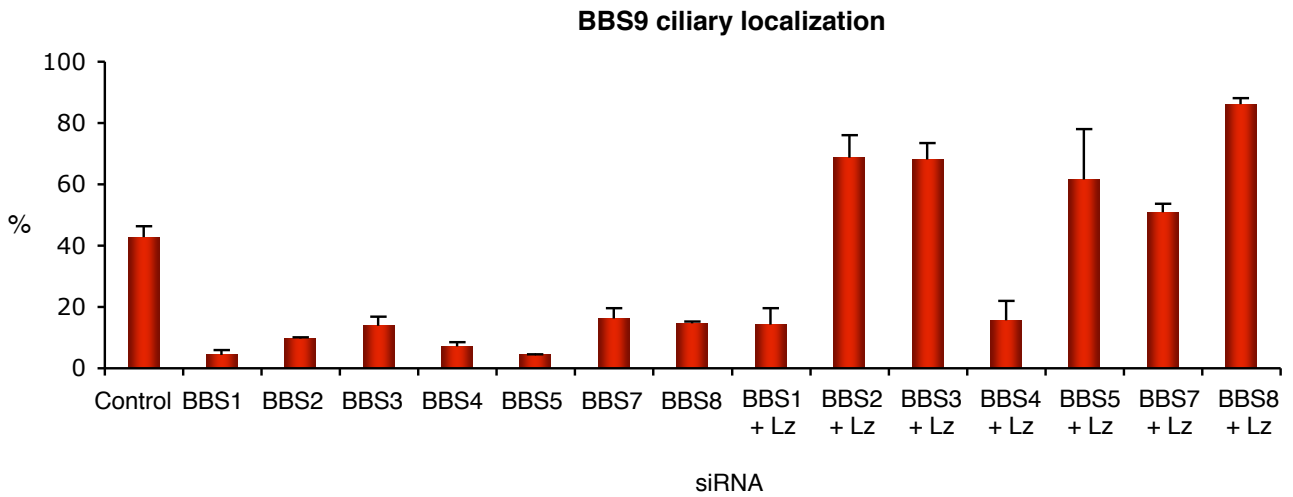

Supplement: Figure S7 — Reduction of LZTFL1 activity restores BBSome ciliary trafficking in BBS3 and BBS5 depleted cells. (A) Localization of BBS8 was probed by immunofluorescence after transfecting indicated siRNAs into hTERT-RPE1 cells. Cilia are labeled with antibodies for acetylated tubulin and γ-tubulin (green) and BBS8 is in red. (B) RPE1 cells were transfected with indicated BBS and LZTFL1 (Lz) siRNAs and BBS8 localization (red) was examined. (C) BBS9 localization (red) was examined after depleting BBS genes and LZTFL1. Note that some panels are also shown in Figure 6. Scale bar, 10 µm. (D,E) Summary of BBSome ciliary localization. Graphs represent average percentages of BBS8 (D) and BBS9 (E) positive cilia from at least two independent experiments with minimum 100 cells counted in each experiment. Error bars represent standard errors. (PDF) [file pgen.1002358.s007.pdf]

Figure S8

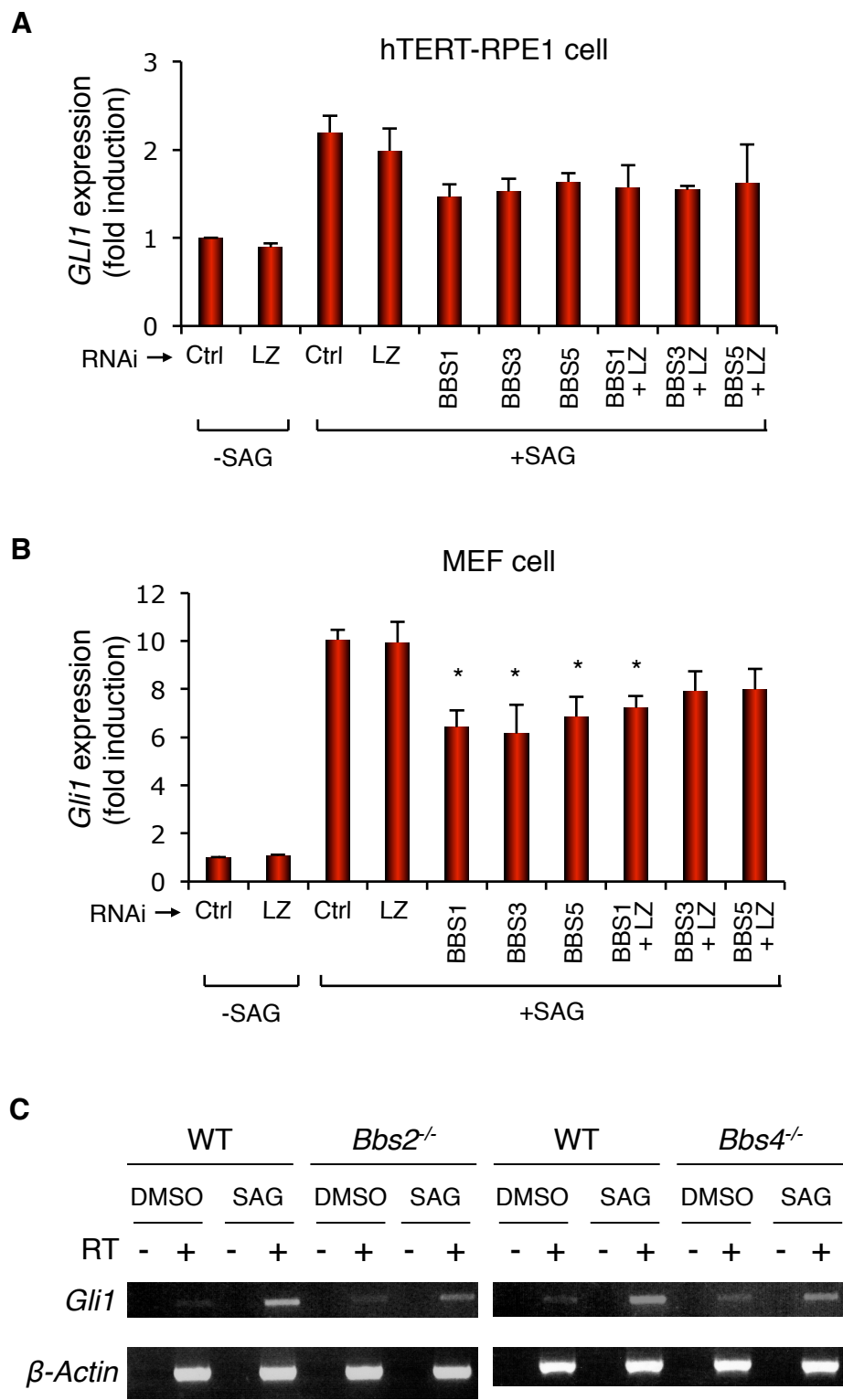

Supplement: Figure S8 — Expression of HH target gene GLI1 upon BBS protein depletion. (A) Expression of GLI1 in hTERT-RPE1 cells. RPE1 cells were transfected with siRNAs as indicated and treated with or without 100 nM SAG for 18 hrs. GLI1 mRNA levels were measured by quantitative PCR. Data are shown as means ± SEM of two independent experiments. (B) Expression of Gli1 in MEF cells. Immortalized MEF cells were transfected with siRNAs as indicated and SAG treatment was performed as in (A). Data are shown as means ± SEM of three independent experiments. Asterisks indicate statistically significant differences compared to control KD cells with SAG treatment (t-test; P<0.05). (C) Reductions in Gli1 expression in Bbs2−/− and Bbs4−/− MEF cells upon SAG treatment. Gli1 expressions in Bbs2−/− and Bbs4−/− MEF cells were compared with that of WT MEF cells from the same liter. Reverse transcription (RT) reactions without reverse transcriptase (-) were used as a negative control. Shown are representative results from a semi-quantitative PCR experiment. (PDF) [file pgen.1002358.s008.pdf]
