# Supplementary material for: A Novel Protein LZTFL1 Regulates Ciliary Trafficking of the BBSome and Smoothened
Source: PLoS Genet. 2011 Nov 3;7(11):e1002358. doi: 10.1371/journal.pgen.1002358 (PMC3207910; doi:10.1371/journal.pgen.1002358)

**Figure S2**

**A**

```
H.sapiens         1                                                     MAELGLNEHHQNEVINYMRFA  21
M.musculus        1                                                     MAELGLNEHHQNEVINYMRFA  21
G.gallus          1 MQAAANRRAARALARLRSPWQRPACSLNRPNRLLRCRVAMAELGLNEHHQNEAINYMRFA  60
X.tropicalis      1                                 MSATDRTAVKIGEFLQKADLGLNDHHQNEVINYIHFA  37
D.rerio           1                                                     MADFGFNEHHQHEVINYMRFA  21
C.intestinalis    1                                                     MSGLGLNDHHHQQAVNFIRFC  21
C.reinhardtii     1                                                      MSIQLSEDGELQVQGYLRFA  20
                                                                             :    :  ::::*:

H.sapiens        22 RSKRGLRLKTVDSCFQDLKESRL-VEDTFTIDEVSEVLNGLQAVVHSEVESELINTAYTN  80
M.musculus       22 RSKRGLRLKTVDSCFQDLKDSRL-VEETFTIDEVSEVLNGLQAVVHSEVESELINTAYTN  80
G.gallus         61 RFKRGLCLKTVDSCFQDLKDSRL-VEETFTVDEVADMLDGLQTIVHSEVESELINTTYTN 119
X.tropicalis     38 RSRRALRLKTVDSCFQDLKDSRL-VEETFTVDEVAEMLQGLQAVVHGEVELELINSAHTN  96
D.rerio          22 RSKRALRLKTVDSCFQDLKESRL-IEETYTVDEVTDILNGLQVLVRGEVEMELINTAHTN  80
C.intestinalis   22 RYKRNQRLKAVDHCFQDLKDSRL-YEDTYTTDEVTEMLDGLLSVVRGEMEAELLNASHTS  80
C.reinhardtii    21 KLKRDQHVREVVSTINDFKADHLRAGDMYNYKELTQMFTELADDTKKLMDKEIQNAYHTN  80
                    : :*    :: *  : :* *  :*   : ::  *:: :    *    :  :: *: *:  *

H.sapiens        81 VLLLRQLFAQAEKWYLKLQTDISELENRELLEQVAEFEKAEITSSNKKPIL-DVTKPKLA 139
M.musculus       81 VLLLRQLFSQAEKWYLKLQTDISELENRELLEQVAEFEKAEFVSSSKKPII-DITKPKLV 139
G.gallus        120 VLLLRQLFSQAEKWYLKLQTDISELENRELLEQVAEFEKSEFTSSNKKPSA-ELIKPKLA 178
X.tropicalis     97 FLLLRQLFMQAEKWYLKLHTDISELENRELLEQVAEFEKAEFSSSNKKANT-EMIKPKLA 155
D.rerio          81 VLLLRQLFSQAEKFYLRLQTDISELENRELLEQVAEFEKTDFKP-NIKANQ-ETTKPKLA 138
C.intestinalis   81 VLQLRQILSQAEKWHLKLCSDISELENRELLEQIADFEEQELSLSGSSD---FKPLPKLE 137
C.reinhardtii    81 ALLVKILLSQAQAQGLELAVDTNSLENEFLLKQISSSEATALSRPASDFVRRNTQLGRLG 140
                      * :: :   **:   * *  *   ***  ** *::  *                  :*

H.sapiens       140 PLNEGG-TAELLNKEILRLQEENEKLKSRLKTIEIQATNALDEKSKLEKALQDLQLDQGN 198
M.musculus      140 PINEGG-TTELLNKEILRLQQENEKLKSRLKTIEIQAVNALDEKSKLERVLQDLQLDQEN 198
G.gallus        179 PINEG--GSELLNKTVACLQEENEKLKSRLKTIETQATAALDEKSKLEKSLKDLQMIQGD 236
X.tropicalis    156 PLFESG-PSELLNKEISRLQEENDKLKARLKTIEYQATSALGDKIKAESALKDLQKVQDN 214
D.rerio         139 PLNEGG-VSELLQREISRLQEDNDKLKARLRTLESRAMCALEDKSKAETALKDLQKSQGV 197
C.intestinalis  138 PLNESG-GVALLQMEIERLKEENERLNERSRSFEKHATKCVEDNKAMQKQIKELG---AK 193
C.reinhardtii   141 TVATVATQDPAVVRERDALRAELQEAKERMAKLQEETTKVMRDRTTLNNQLNSLRDELAA 200
                      :        :       *: :       *     :   :   : :       :   *

H.sapiens       199 QKDFIKAQD------LSNLENTVAALKSEFQKTLNDKTENQKS-----LEENLATAKHDL 247
M.musculus      199 QQDLLKAQD------LDDLENTVATLRSEFQKTLNDKTENQKS-----LEENLAAAKHDL 247
G.gallus        237 QK-TDVNKD------IVELENKVAALKCQFEKTLNDSTANQKF-----LEENLVTTKHDL 284
X.tropicalis    215 QKAKQNTQD------INILEKTMADLKCDFEKTVNDHTVSQKY-----LEDNLASTKHEL 263
D.rerio         198 HQSVMCAQE------IANLEDTVAAMQADFEKTLNTNITSQKD-----LQDSLVSTKHDL 246
C.intestinalis  194 PVAAVDNSS------VSELETKLKAMTADFSESMDSKSALNEA-----LAADLTSTKHDL 242
C.reinhardtii   201 KDTALRAALGDKEAAVAGLSNKMAALEAAGRGGVSAEAAAALEKKAGGLGGQLAAVQEEL 260
                                  :  *    :  : :     :                *   * : : :*

H.sapiens       248 LRVQEQLHMAEK-------ELEKKFQQTAAYRNMKEILTKKNDQIKDLRKRLAQYEPE-- 298
M.musculus      248 LRVQEQLSMAEK-------ELEKKFQQTAAYRNMKEILTKKNDQIKDLRKRLAKYESE-- 298
G.gallus        285 LKVQDQLSTAEK-------ELEKKFQQTAAYRNMKEILTKKNEQIKDLRRRLSKYEPE-- 335
X.tropicalis    264 LRVQEQLSSAEK-------ELEKKFQQTAVYRNMKDILTKKNDQIKDLRKRLQKYEPE-- 314
D.rerio         247 LRVQGQLSLAEK-------ELEKKFQQTAAYRNLKEILNKKNEQIKDLRKRLLRYESA-- 297
C.intestinalis  243 LKVQEQLEMAEK-------DLEKKFRETGAFRNLRDMLAKKNEQLKELRKKLGKYEQT-- 293
C.reinhardtii   261 QAARNQLALKDKELRAASEALSGKLQESKQFLAMKQMMQSKSQEAAALRKRLEQYEPQSV 320
                       : **    :*         *   * :::  :  :: ::  *   :     **::* :**

H.sapiens       299 ---D    299
M.musculus      299 ---D    299
G.gallus        336 ---D    336
X.tropicalis    315 ---D    315
D.rerio         298 ---E    298
C.intestinalis  294 ---D    294
C.reinhardtii   321 PSADTA  326
                       :
```

**Figure S2 (Continued)**

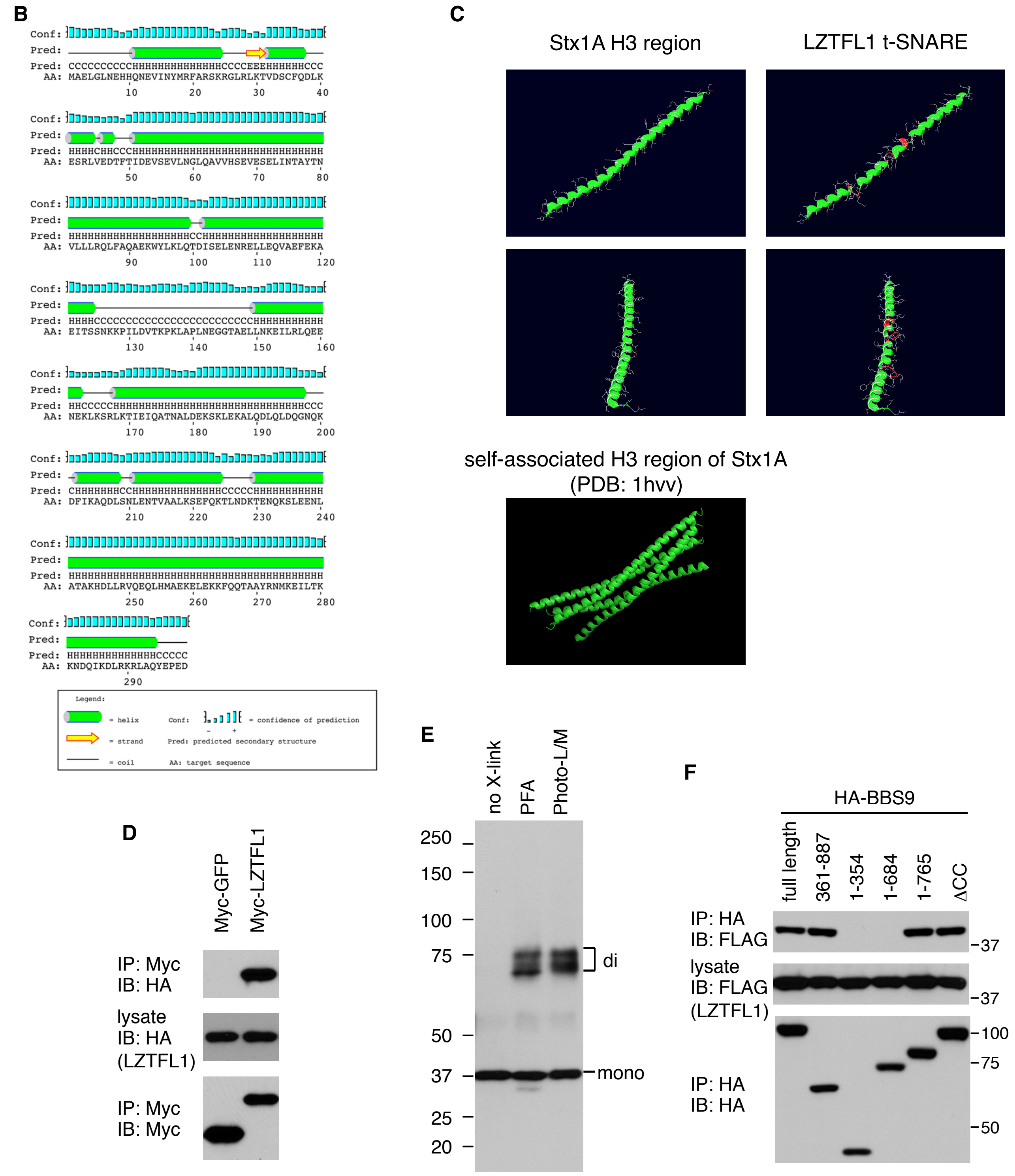

Supplement: Figure S2 — LZTFL1 structure and interaction. (A) Amino acid sequence alignment of LZTFL1 homologs from human (Homo sapiens), mouse (Mus musculus), chicken (Gallus gallus), frog (Xenopus tropicalis), zebrafish (Danio rerio), Ciona intestinalis, and Chlamydomonas reinhardtii. The blue line above the amino acid (aa) sequences represents the predicted coiled-coil domain following [16]. The red line above the aa sequences designates the t-SNARE domain determined by PSI-BLAST. Red boxes mark the basic amino acids mutagenized in the KR (to AS) construct (see Figure 2). Asterisks represent residues conserved in all species and colons, similar residues. (B,C) Prediction of LZTFL1 secondary structure (B) and modeling of C-terminal t-SNARE domain of LZTFL1 (C). Rat Stx1A H3 domain (PDB: 1hvv, chain A) was used as a template and aligned with human LZTFL1 aa 212–284. Lower panels are 90° rotated images of the upper panels. (D) Homo-oligomerization of LZTFL1. HA-LZTFL1 was co-transfected with Myc-GFP or Myc-LZTFL1 into HEK293T cells and IP was performed with anti-Myc antibody. (E) HEK293T cells were cross-linked with 1% paraformaldehyde (PFA) in PBS or with L-Photo-Leucine and L-Photo-Methionine (Photo-L/M). Lysates were analyzed by SDS-PAGE and immunoblotting. Monomeric (mono) and dimeric (di) forms of LZTFL1 were marked. Several forms of dimeric LZTFL1 were found. (F) LZTFL1-interaction domain mapping in BBS9. Deletion mutants of HA-BBS9 were co-transfected with FLAG-LZTFL1. ΔCC indicates deletion of the coiled-coil domain (amino acids 378–408). (PDF) [file pgen.1002358.s002.pdf]
