# Supplementary material for: A Novel Protein LZTFL1 Regulates Ciliary Trafficking of the BBSome and Smoothened
Source: PLoS Genet. 2011 Nov 3;7(11):e1002358. doi: 10.1371/journal.pgen.1002358 (PMC3207910; doi:10.1371/journal.pgen.1002358)
Supplement: Table S1 — Summary of mass spectrometry analysis of the LAP-BBS4 eluate. (DOCX) [file pgen.1002358.s009.docx]

**Table S1. Summary of mass spectrometry analysis of the LAP-BBS4 eluate.**

| MW (kDa) | Protein | Unique Peptides Detected | Peptide Coverage (%) |
| --- | --- | --- | --- |
| 99.0 | Bbs9 | 15 | 13.8 |
| 80.3 | Bbs7 | 30 | 29.8 |
| 79.9 | Bbs2 | 19 | 15.1 |
| 65.1 | Bbs1 | 20 | 36.4 |
| 58.4 | Bbs8 | 10 | 12.7 |
| 38.9 | Bbs5 | 10 | 28.5 |
| 34.8 | Lztfl1 | 6 | 18.1 |
