# Supplementary material for: A Novel Protein LZTFL1 Regulates Ciliary Trafficking of the BBSome and Smoothened
Source: PLoS Genet. 2011 Nov 3;7(11):e1002358. doi: 10.1371/journal.pgen.1002358 (PMC3207910; doi:10.1371/journal.pgen.1002358)
Supplement: Table S2 — Summary of mass spectrometry analysis of the FS-LZTFL1 eluate. (DOCX) [file pgen.1002358.s010.docx]

**Table S2. Summary of mass spectrometry analysis of the FS-LZTFL1 eluate.**

| MW (kDa) | Protein | Unique Peptides Detected | Peptide Coverage (%) |
| --- | --- | --- | --- |
| 99.3 | BBS9 | 4 | 4.7 |
| 80.4 | BBS7 | 12 | 17.5 |
| 80.0 | BBS2 | 8 | 9.4 |
| 41.8 | FS-LZTFL1 | 19 | 62.5 |
| 34.6 | LZTFL1 | 16 | 51.5 |
