# Supplementary material for: A Novel Protein LZTFL1 Regulates Ciliary Trafficking of the BBSome and Smoothened
Source: PLoS Genet. 2011 Nov 3;7(11):e1002358. doi: 10.1371/journal.pgen.1002358 (PMC3207910; doi:10.1371/journal.pgen.1002358)
Supplement: Table S3 — Quantitative real-time PCR primer sequences. (DOCX) [file pgen.1002358.s011.docx]

**Table S3. Quantitative real-time PCR primer sequences.**

| **Name** | **Sequence** |
| --- | --- |
| **F-hBBS1** | GCCATCCTGACCATGAACCTC |
| **R-hBBS1** | AAAGCAAAGGCTGGTCACTGC |
| **F-hBBS2** | GGGAGATTACCGGATGGATGG |
| **R-hBBS2** | GCACTGGTGTCCATGAGGTTG |
| **F-hBBS3** | CAGGCATCCTTCCCATGTAGG |
| **R-hBBS3** | GAGCTGGCAACAGGAATGGAC |
| **F-hBBS4** | GCAGCCTCTGGGCTCTAATCA |
| **R-hBBS4** | TGGCTCCAGAGGAAGAGATGG |
| **F-hBBS5** | TCCACGGCTGTGGAGAGAT |
| **R-hBBS5** | CCAGGTCTTGTTTTCATTTGC |
| **F-hBBS7** | TCCTGCCTCAAGACACAGAGC |
| **R-hBBS7** | TGATAACCCCTCCCAGTTCCA |
| **F-hBBS8** | ACTTGGGACATGTAGCTGTGG |
| **R-hBBS8** | GTAATAGTGCCCTTGCCTGTTC |
| **F-hBBS9** | GGCCCATGACTGAGAGAGAAG |
| **R-hBBS9** | CACTCTGTTCTGCAGTGTGACC |
| **F-hLZTFL1** | CCCCATTTTCTGTGGTCCAAC |
| **R-hLZTFL1** | AGCCTAAAGGAACGGGAGAGG |
| **F-hRPL19** | CCGGAAGCTCATCAAAGATGG |
| **R-hRPL19** | TTGGCTGTACCCTTCCGCTTA |
| **F-hGLI1** | CTGCTCCAGCTAGAGTCCAGA |
| **R-hGLI1** | GACAGAGGTTGGGAGGTAAGG |
| **F-mBbs1** | CAAACACCCCAAGTACTGCAT |
| **R-mBbs1** | TGAAGCCATGCAGACTCTCTT |
| **F-mBbs3** | ACGGAGGCATAGAGAGGAAG |
| **R-mBbs3** | CCCATATCGCGGCTTAAATA |
| **F-mBbs5** | CCTCGGTCACCATGTCTGT |
| **R-mBbs5** | CCGGGTCTTGTTTTCATCTG |
| **F-mGli1** | ACTAGGGGGCTACAGGAGGA |
| **R-mGli1** | ACCTGGACCCCTAGCTTCAT |
| **F-mLztfl1** | GGCTGGTGGAGGAGACTTTCA |
| **R-mLztfl1** | CTGCCGTAGGAGCAGGACATT |
| **F-mRpl19** | GCAAGCCTGTGACTGTCCATT |
| **R-mRpl19** | GCATTGGCAGTACCCTTCCTC |
